# Molecular Evolution of Classic Human Astrovirus, as Revealed by the Analysis of the Capsid Protein Gene

**DOI:** 10.3390/v11080707

**Published:** 2019-08-01

**Authors:** Nan Zhou, Lu Zhou, Bei Wang

**Affiliations:** 1 Key Laboratory of Environmental Medicine and Engineering of Ministry of Education, Department of Epidemiology and Statistics, School of Public Health, Southeast University, Nanjing 210009, Jiangsu, China; sduzhounan@163.com; 2 Jiangsu Provincial Center for Disease Control and Prevention, Nanjing 210009, Jiangsu, China; zhoulu1212@163.com

**Keywords:** astrovirus, capsid, evolution

## Abstract

Classic human astroviruses (HAstV) are major global viral agents for gastroenteritis, but the molecular characteristics of classic HAstVs are not well understood. Here, we presented the molecular evolution of all classic HAstV serotypes by the analysis of the capsid protein sequences. Our results show that classic HAstVs can be divided into four groups with the most recent common ancestor (TMRCA) of 749. The overall evolutionary rate of classic HAstVs on the capsid gene was 4.509 × 10^−4^ substitutions/site/year, and most of the serotypes present a clock-like evolution with an amino acid accumulation of mutations over time. The mean effective population size of classic HAstVs is in a downward trend, and some positive and more than 500 negative selection sites were determined. Taken together, these results reveal that classic HAstVs evolve at the intra-serotype level with high genetic heterogeneity and are driven by strong purifying selection. Long-term surveillance of classic HAstVs are needed to enrich the genomic data for further analysis.

## 1. Introduction

Astroviruses are non-enveloped, positive sense, single-stranded RNA viruses [1]. Their genome is 6.8–7.9 kb in length and consists of three open reading frames (ORFs), designated ORF1a, ORF1b, and ORF2. ORF1a and ORF1b, at the 5′ end of the genome, encode nonstructural proteins, including the RNA-dependent RNA polymerase (RdRp), while ORF2, at the 3′ end, encodes the capsid protein precursor [2]. Astroviruses are classified into the genera *Mamastrovirus* and *Avastrovirus* [3] and can infect various hosts from birds to mammals, including humans [4]. Human astroviruses (HAstVs) were first recognized in children stool samples with diarrhea in 1975 [5]. Since then, HAstVs have been the well-established viral agents of gastroenteritis globally, and the number of astrovirus-related publications increase steadily [6].

In recent years, two novel astroviruses clades, namely, Melbourne (MLB) and Virginia/Human-Mink-Ovine-like (VA/HMO), emerged and have been detected in stool samples from humans with gastroenteritis worldwide [7,8,9]. However, the association between novel HAstVs and gastroenteritis is to be confirmed. The overall detection rate of novel HAstVs in stool was lower [10], and classic HAstVs (classified into eight serotypes: HAstV-1 to HAstV-8) are still the second or third most common viral agents responsible for gastroenteritis in young children [11]. Serum antibodies to at least one classic HAstV serotype were detectable in 90% of children by the age of 5 years [6]. In the US, approximately 10% of reported acute gastroenteritis outbreaks in childcare centers were caused by classic HAstVs [12].

The global disease burden of classic HAstVs is high. However, owing to the unavailablity of cell culture systems and robust small animal models, classic HAstVs are among the least studied enteric RNA viruses [6]. Happily, with the development of bioinformatics technologies, molecular analysis using the sequences from public databases has been successfully conducted in various viruses, like norovirus, rotavirus and influenza virus [13,14,15]. It is becoming valuable to elucidate the molecular characteristics of viruses from genomic data for epidemic prediction and management. But the molecular analysis of classic HAstVs is inadequate. Here, in order to gain a better understanding of the molecular characteristics of classic HAstVs, we presented a comprehensive description of the molecular evolution of classic HAstV serotypes by analyzing the complete capsid gene for all strains.

## 2. Materials and Methods

### 2.1. Dataset

In order to obtain classic HAstV ORF2 sequences, we searched the corresponding taxonomy ID of HAstV (Taxonomy ID: 1868658) in NCBI’s GenBank Database. Interrogation of the database was terminated on March 2019, and the isolated time and location for each strain were retrieved from the GenBank database or the associated publications. The complete or nearly complete sequences (nucleotide position 4328 to 6691 according to HAstV-1/Oxford-1/1993/UK, GenBank accession No. L23513) were selected to analyze the characteristics of molecular evolution.

### 2.2. Genetic Diversity Analysis

Multiple alignment was performed by ClustalW as implemented in MEGA v7.0.26 [16], and the recombination event was determined by the Recombination Detection Program (RDP) v4.56 with *p*-value < 0.05 in 3 or more methods [17]. The nucleotide and amino acid identities were calculated using BioEdit 7.1.3.0 [18]. The inter-serotype mean amino acid distance was estimated based on Poisson model by MEGA v7.0.26 [16].

### 2.3. Root-to-Tip Divergence Analysis

The root-to-tip divergence was calculated by TempEst v1.5 [19] based on the inferred maximum likelihood (ML) tree which was constructed using MEGA v7.0.26 [16]. The best-fitting root option was selected to ensure the best correlation of the root-to-tip divergence. Then the root-to-tip divergence against the isolation year of each sequence was plotted and visualized to evaluate the evolutionary clock-like nature of classic HAstVs.

### 2.4. Accumulation Pattern of Amino Acid Substitutions

In order to visualize the accumulation of amino acid substitutions over time, we calculated the pairwise amino acid difference by MEGA 7.0.26 [16] among sequences with the same serotype. Then, the mean amino acid difference was calculated with the same time-span of isolation for each serotype. Finally, the mean amino acid difference and the time-span of isolation was plotted and visualized to evaluate whether amino acid substitution accumulated over time. The fitting line was also estimated to discuss the possible linear accumulation and the accumulative rate.

### 2.5. Evolutionary Analysis

We used the Bayesian Markov Chain Monte Carlo (MCMC) method in BEAST package v1.8.3 to estimate the time-scale maximum clade credibility (MCC) tree and evolutionary rate (nucleotide substitutions/site/year) of all complete classic HAstV ORF2 sequences [20]. Briefly, the best-fit nucleotide substitution model was estimated by IQ-TREE web server on the basis of corrected Akaike’s Information Criterion (AICc) score [21]. Three clock models (strict clock, uncorrelated lognormal relaxed clock, and uncorrelated exponential relaxed clock) and Bayesian skyline coalescent tree were selected and compared by Akaike’s Information Criterion through MCMC (AICM) [22] using Tracer v1.6 (http://tree.bio.ed.ac.uk/software/tracer/), and the model with the lowest AICM value was used. The convergence of parameters was evaluated using Tracer v1.6 and an effective sample size value >200 was considered acceptable. The MCC tree was obtained after 10% burn-in using TreeAnnotator v1.8.2 and visualized by FigTree v1.4 [23]. The Bayesian skyline plot (BSP) of all complete classic HAstV ORF2 sequences was constructed using Tracer 1.6. Furthermore, according to the aforementioned methods, the evolutionary rate and Bayesian skyline plots of HAstV-1, -3, -4 and -5, which have more than 10 sequences, were also estimated.

### 2.6. Selection Pressure Analysis

The selection pressure on the capsid gene of classic HAstVs was evaluated by estimating the nonsynonymous (dN) and synonymous (dS) substitutions ratio (dN/dS) using the Datamonkey server [24]. The site under positive selection (dN>dS) was determined with a *p*-value threshold of 0.1 using the single-likelihood ancestor counting (SLAC), fixed effects likelihood (FEL) and mixed effects model of evolution (MEME) methods. The site under negative selection (dN < dS) was determined with a *p*-value threshold of 0.1 using the SLAC and FEL methods.

## 3. Results

### 3.1. Description of Classic HAstV ORF2 Sequences in the GenBank Database

Excluding strains cultivated in eukaryotic cells and from environmental samples, a total of 1111 partial or complete capsid sequences of classic HAstVs with geographical and temporal information were obtained (Supplemental Table S1). These sequences were identified in 30 countries, and most of them were from Europe and Asia since 2005 (63.3%, 703/1111). Of these, HAstV-1 was the most dominant serotype (76.7%, 852/1111), followed by HAstV-4 (7.3%, 81/1111), HAstV-5 (4.9%, 54/1111), HAstV-3 (4.8%, 53/1111) and HAstV-8 (3.4%, 38/1111).

### 3.2. Genetic Diversity of Classic HAstV ORF2 Sequences

After excluding the possible recombination sequences determined by RDP 4.5.6, a total of 116 complete (or nearly complete, 2316–2340 bp) capsid sequences of classic HAstVs were retrieved from the GenBank database and analyzed for molecular evolution in this study (Supplemental Table S2). These sequences were isolated from 1971 to 2015, and all serotypes had a range of collection years more than 15. The nucleotide and amino acid similarity of the sequences ranged from 58.8 to 100% and 57.5 to 100%, respectively (Table 1). HAstV-2 and -4 had a higher amino acid distance when compared with other types. The minimum inter-serotype mean amino acid distance was between HAstV-3 and HAstV-7 (0.167, Table 1), and the maximum inter-serotype mean amino acid distance was between HAstV-4 and HAstV-7 (0.431, Table 1).

### 3.3. Root-to-Tip Divergence Analysis

**Figure 1:**
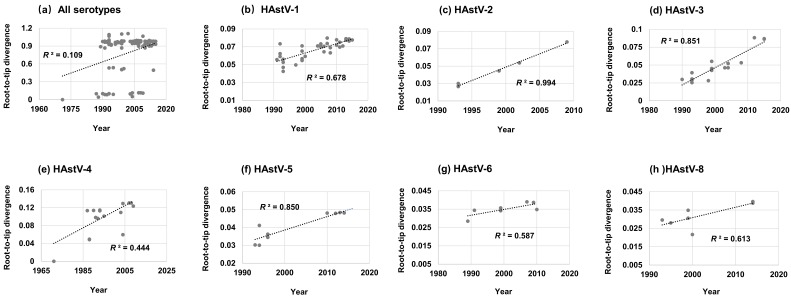
Root-to-tip divergence plots of the capsid protein gene of classic HAstVs: (**a**) all serotypes, (**b**) HAstV-1, (**c**) HAstV-2, (**d**) HAstV-3, (**e**) HAstV-4, (f) HAstV-5, (**g**) HAstV-6, and (**h**) HAstV-8. The *Y*-axis shows the root-to-tip divergence based on the maximum likelihood tree, and the *X*-axis indicates the isolation year. Each sequence is represented by a circle, and the dashed line indicates a linear regression line of the root-to-tip divergence and isolation year.

To investigate the evolutionary clock-like nature of classic HAstVs on the ORF2 region, the root-to-tip divergence plots were conducted based on the inferred ML trees. The results showed that classic HAstVs evolved with a poor clock-like signal with a coefficient of determination (R^2^) value of 0.109 (Figure 1a). The root-to-tip divergence for each serotype was also analyzed except for HAstV-7, which has only three sequences and could not allow us to infer an ML tree by bootstrapping with 1000 times. The plots (Figure 1b–h) revealed that classic HAstVs presented a linear evolution at the intra-serotype level. HAstV-2, -3 and -5 presented a stronger clock-like evolution, with R^2^ values of 0.994, 0.851 and 0.850, respectively. Nevertheless, HAstV-1, -4, -6 and -8 presented a moderate clock-like pattern with R^2^ values of 0.678, 0.444, 0.587 and 0.613, respectively.

### 3.4. Accumulation Pattern of Amino Acid Substitutions

**Figure 2:**
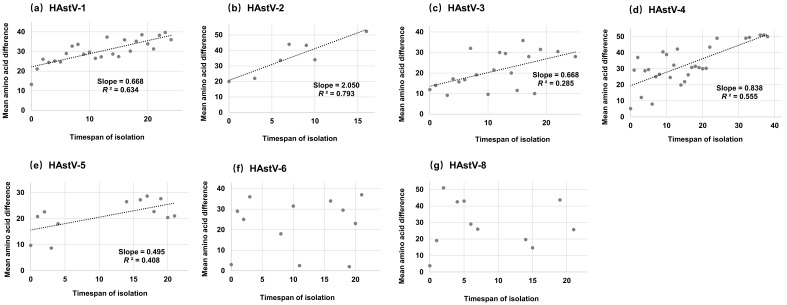
Accumulation plots of amino acid over time of classic HAstVs capsid protein gene: (**a**) HAstV-1, (**b**) HAstV-2, (**c**) HAstV-3, (**d**) HAstV-4, (**e**) HAstV-5, (**f**) HAstV-6, and (**g**) HAstV-8. The *X*-axis indicates the time-span of isolation, and the *Y*-axis shows the mean amino acid difference. The dashed line indicates a linear regression line of the mean amino acid difference and time-span of isolation (HAstV-6 and -8 presented no linear signal, and the fitting line was not performed).

We developed an algorithm to evaluate the relationship between amino acid diversity and time-span among strains from a given serotype (HAstV-7 was also not analyzed in this part because the number of sequences was relatively small). Firstly, the pairwise amino acid differences were calculated and averaged according to the time-span of isolation for each serotype. Afterwards, the algorithm generated a diagram in which the mean amino acid difference plotted against the timespan of isolation. Our results showed that the mean pairwise amino acid difference of the capsid protein from HAstV-6 and HAstV-8 were not influenced by the time-span of isolation (Figure 2f,g). Nevertheless, the mean amino acid difference of the capsid protein from other serotypes accumulated continually over time (Figure 2a–e), and HAstV-1, -2, -4 and -5 sequences presented moderate linear accumulation (R^2^: 0.408–0.793). In addition, the amino acid substitutions of HAstV-2 accumulated faster than other serotypes (Slope = 2.050), and HAstV-5 owned the slowest accumulative rate (Slope = 0.495).

### 3.5. Time-Scale Phylogenetic Tree

**Figure 3:**
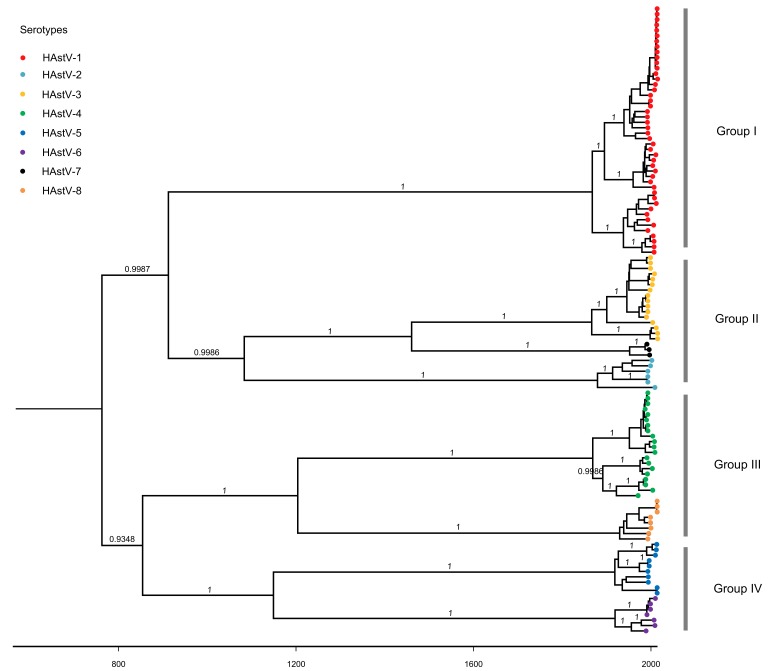
Phylogenetic tree of the capsid protein gene of classic HAstVs constructed by the Bayesian Markov Chain Monte Carlo method. Branches are scaled in time and sequences are colored by serotypes. Posterior probability support is indicated by the number along the branch.

The time-scale phylogenetic tree of the complete ORF2 sequences of classic HAstVs was constructed using the Bayesian MCMC method (Figure 3), which was in a balanced branching pattern and showed that classic HAstVs can be divided into four groups. Group I only contained one serotype (HAstV-1), and the other groups contained more than two serotypes (Group II: HAstV-2, -3 and -7; Group III: HAstV-4 and -8; Group IV: HAstV-5 and -6). The most recent common ancestor (TMRCA) of the tree was around 749 (95% highest posterior densities [HPDs]: 457–1017). The years of divergence of HAstV-1, -2-, 3 and -4 were similar (HAstV-1: 1866, 95% HPDs: 1832–1896; HAstV-2: 1878, 95% HPDs: 1844–1907; HAstV-3: 1865, 95% HPDs: 1829–1898; HAstV-4: 1867, 95% HPDs: 1836–1896), and HAstV-5 and -6 diverged nearly at the same time (HAstV-5: 1918, 95% HPDs: 1895–1937; HAstV-6: 1918, 95% HPDs: 1897–1938). The ancestor of HAstV-7 and -8 diverged later than other serotypes (HAstV-7: 1951, 95% HPDs: 1938–1965; HAstV-8: 1929, 95% HPDs: 1909–1945).

### 3.6. Evolutionary Rate of ORF2 Sequences

The evolutionary rate was estimated only for serotypes presenting more than 10 sequences (HAstV-1, -3, -4, -5 and all serotypes). The selected model was described in Supplemental Table S3. The results (Table 2) showed that the overall evolutionary rate of classic HAstVs on the *ORF2* gene was 4.509 × 10^−4^ substitutions/site/year (95% HPDs: 3.558 × 10^−4^–5.512 × 10^−4^ substitutions/site/yea). HAstV-3 had a higher evolutionary rate (2.195 × 10^−3^ substitutions/site/year), and the evolutionary rates of HAstV-1 and -5 were similar (7.898 × 10^−4^ vs. 7.577 × 10^−4^, Table 2). Besides, a higher ratio of substitutions rate at the third codon compared with the first/second codon positions was also observed (Table 2).

### 3.7. Phylodynamics of Classic HAstVs Strains

**Figure 4:**
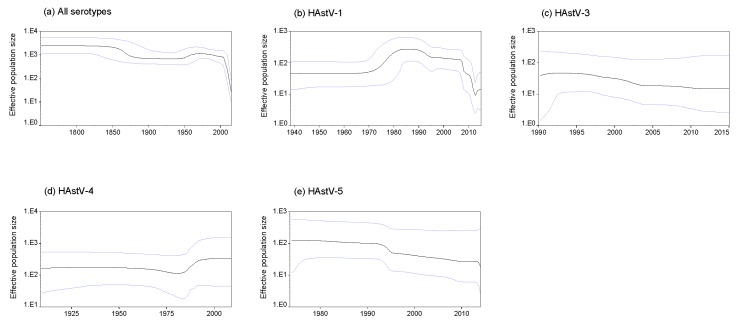
Bayesian skyline plots of the capsid protein gene of classic HAstVs: (**a**) all serotypes, (**b**) HAstV-1, (**c**) HAstV-3, (**d**) HAstV-4, and (**e**) HAstV-5. The *Y*-axis shows the effective population size. Mean effective population size is shown as a black line. The 95% highest posterior densities are shown as light blue lines.

The Bayesian skyline coalescent model was selected as the tree in this study to evaluate the changes in the effective population size of classic HAstVs on the *ORF2* gene. On the whole, the effective population sizes of classic HAstVs have actually fallen and descended rapidly around 2000 (Figure 4a). For each serotype, the mean effective population sizes of HAstV-1 remained unstable and presented drastic changes in the past 50 years, which began to grow around 1975 and reached the peak around 1985. After that, it decreased slowly and presented a sharp fall from 2005 to 2012. Subsequently, it began growing again (Figure 4b), whereas the mean effective population sizes of HAstV-3 and -5 were in a slow decline (Figure 4c,e). The mean effective population size of HAstV-4 decreased slightly around 1975 and began to increase from 1985 to 1995, then remained constant (Figure 4d).

### 3.8. Selective Pressure Analysis

To investigate the selective pressure on each site in the capsid gene of classic HAstVs, we calculated the ratio of nonsynonymous to synonymous substitution. The mean dN/dS value was 0.142, and the SLAC and FEL methods both recognized more than 500 negative selected sites. Additionally, the SLAC and FEL methods identified two (amino acid position at 52 and 663) and nine sites (amino acid position at 4, 21, 52, 55, 57, 659, 663, 742 and 804) under positive selection, respectively. Up to 28 positively selected sites (amino acid position at 4, 21, 52, 55, 57, 60, 72, 492, 567, 659, 663, 668, 697, 701, 707, 729, 742, 780, 781, 782, 796, 797, 798, 800, 804, 806, 807 and 808) were detected by the MEME method.

## 4. Discussion

Classic HAstVs are the leading viral agents for gastroenteritis. However, the molecular evolution of classic HAstVs has not been discussed in detail. A complete sequence can provide us with more detailed information about the molecular evolution. In this study, a large number of the capsid protein sequences (complete or nearly complete) of classic HAstVs in the GenBank database were retrieved and analyzed to obtain a picture of their molecular characteristics.

A root-to-tip divergence analysis was conducted in this study, which is an approach to explore the clock-like manner of the evolution [25,26]. It has been reported that norovirus non-GII.4 genotypes presented a linear evolution at the intra-variant level, and the emergence of a novel norovirus GII.17 variant in 2014–2015 was speculated to be caused by this evolution pattern [26]. Classic HAstVs can also be classified into many variants for each serotype [27]. Nevertheless, our results show that classic HAstVs present a linear evolution at the intra-serotype level, indicating that each serotype of classic HAstVs evolves as a whole. Recombination and mutation are the main factors determining the molecular evolution of RNA viruses [28,29]. Recombination events of classic HAstVs were often identified at the ORF1b–ORF2 junction, which can contribute to the acquisition of a novel polymerase and change the evolution of the capsid protein [30,31,32,33]. The co-evolution of classic HAstVs at the intra-serotype level may also suggest that polymerase types have a minimal impact on the long-term clock-like evolution of the capsid protein of classic HAstVs, just like non-GII.4 norovirus [26].

Previous research by Parra et al. identified two different evolutionary patterns of norovirus: evolving and static. The evolving genotype (represented by the GII.4) presents amino acid accumulation of mutations over time, whereas static genotypes do not and have low prevalence due to their highly conserved and possible genetic fragility [34]. Our data show that several serotypes of classic HAstVs including HAstV-1 present an evolving pattern and these serotypes almost have relatively higher prevalence when compared with others [35]. This indicates again that the evolutionary patterns may be a signal to evaluate the relative prevalence of genotypes (or serotypes) in some viruses. In this study, the results of TMRCA for each serotype reveal that these serotypes persisted and co-circulated for a long time in humans. The time-scale MCC tree of the capsid protein gene of classic HAstVs results in four separated groups and serotypes in each group may have a common ancestor. Nevertheless, phylogenetic analysis based on *ORF1a* gene showed that classic HAstVs were only divided into two groups [36,37]. These may indicate that different ORFs of classic HAstVs evolve independently. Furthermore, skewed or ladder-like phylogenetic topology means the repeated occurrences of punctuated immune escape and there is a temporal replacement of predominant variants driven by the immune response of the host, such as norovirus GII.4, influenza H3N2 viruses [38]. In this study, the time-scale MCC tree of classic HAstVs presents as well balanced and lacks significant temporal structures at the variant level for each serotype, which reiterates that classic HAstVs may evolve at the intra-serotype level.

The nucleotide evolutionary rate of positive-strand RNA viruses can range from 10^−9^ to 10^−2^ substitutions/site/year determined by their genome and replication strategies [39,40,41]. A previous study has reported the evolutionary rate of classic HAstVs was approximately 3.7 × 10^−3^ substitutions/site/year based on the genome fragments without recombination breakpoints [42], which was much higher than our estimate (4.509 × 10^−4^ substitutions/site/year). But a limited number of sequences (16 sequences belonging to five serotypes) were included in that study, and we believe that our estimate result is more accurate. In addition, our results reveal that classic HAstVs evolve relatively slower when compared with other gastroenteritis viruses, such as norovirus GI and GII which evolve with similar evolutionary rates of about 10^−3^ substations/sites/year at the capsid level [43,44]. This may partially explain the prevalent discrepancy between norovirus and classic HAstVs, since substitution rates are thought to be associated with the rates of inter-host transmission [39]. To examine the change in the effective population sizes in classic HAstVs, BSP analyses were performed in this study. BSP is the most widely applied demographic inference method, using standard MCMC sampling procedures to predict the relative effective population size over time directly from a sample of gene sequences, where changes in effective population size reflect a change in genetic diversity and help illustrate a demographical history [45,46]. We found that the effective population size of HAstV-1 was growing in the past few years. The effective population size of HAstV-4 was stable after it reached the peak around 1995. Such BSP data may predict that the prevalence of HAstV-1 and HAstV-4 will be still relatively higher in the future.

Next, selection pressure analysis was performed. Selection pressure analysis allows identifying putative sites with positive selection for immune escape. In this study, the discrepancy of detected sites under positive selection by three methods are attributed to the algorithmic models [47,48], and the sites prone to positive selection are located at the C-terminal half of capsid protein, which is similar to a previous report that included at least one sequence from each serotype [49]. This position is deemed to comprise the outer surface of the viral capsid and these positively selected sites may be associated with the residues exposed to the immune pressure or involved in receptor recognition [49,50]. Furthermore, the mean dN/dS value was relatively low, and a large number of negatively selected sites were recognized, revealing that positive selection at the codon level is not the dominant mechanism driving diversity and classic HAstVs are driven by strong purifying selection.

In summary, although the number of sequences analyzed in this study is limited, and selection bias or the origin of sequences (i.e. immunocompromised vs. immunocompetent), which was not provided in each analyzed sequence in the GenBank database, may affect the accuracy of the data, our results still provide valuable information. We found that HAstV-1 and HAstV-4 were the two most predominant serotypes in the GenBank database. Classic HAstVs can be divided into four groups, and most of the serotypes evolve as a whole with a higher evolutionary rate and amino acid accumulation of mutations over time. The mean effective population size of classic HAstVs is in a downward trend, and purifying selection is a dominant force among the capsid genes of classic HAstVs. This study also highlights that genomic sequences from public databases can be analyzed to increase the understanding of the molecular evolution of viruses, and molecular epidemiological study should be enhanced, especially in developing countries, to enrich the genomic databases.

## Figures and Tables

**Table 1:** Summary of the complete capsid sequences of classic human astroviruses (HAstVs) analyzed in this study.

Serotype	No. of Sequences	Years	Duration of Collection Years	Similarity		Inter-Serotype Mean Amino Acid Distance						
				Nucleotide	Amino Acid	HAstV-1	HAstV-2	HAstV-3	HAstV-4	HAstV-5	HAstV-6	HAstV-7
HAstV-1	46	1991–2015	24	89.4–100%	90.9–100%							
HAstV-2	6	1993–2009	16	87.6–99.8%	88.7–99.8%	0.341						
HAstV-3	16	1990–2015	25	88.2–99.7%	92.9–99.8%	0.242	0.305					
HAstV-4	20	1971–2009	38	89.1–99.9%	91.6–100%	0.429	0.394	0.395				
HAstV-5	10	1993–2014	21	93.1–100%	96.0–100%	0.327	0.389	0.305	0.410			
HAstV-6	7	1989–2010	21	93.5–99.8%	95.2–99.8%	0.304	0.366	0.289	0.393	0.254		
HAstV-7	3	1991–1997	16	96.6–99.5%	96.4–99.3%	0.282	0.332	0.167	0.431	0.302	0.288	
HAstV-8	8	1993–2014	21	93.6–100%	91.5–100%	0.320	0.333	0.296	0.314	0.293	0.299	0.329
All	116	1971–2015	44	58.8–100%	57.5–100%							

**Table 2:** Evolutionary rate and ratio of substitution rate at the third codon/first+second codon positions. 95% highest posterior densities [HPDs].

Serotypes	Substitution Rate (Substitutions/Site/Year)		Ratio of Rate (Codon 3/Codon 1 + 2)	
	Mean	95% HPDs ^a^	Mean	95% HPDs
HAstV-1	7.898 × 10^−4^	6.143 × 10^−4^–9.747 × 10^−4^	2.684	2.198–3.396
HAstV-3	2.195 × 10^−3^	7.439 × 10^−4^–3.565 × 10^−3^	4.484	3.374–6.225
HAstV-4	3.964 × 10^−4^	1.655 × 10^−4^–6.014 × 10^−4^	2.769	2.109–3.788
HAstV-5	7.577 × 10^−4^	2.836 × 10^−4^–1.277 × 10^−3^	3.724	2.695–5.485
All	4.509 × 10^−4^	3.558 × 10^−4^–5.512 × 10^−4^	2.727	2.414–3.082

^a^ HPDs, the highest posterior densities.

## Supplementary Materials

The following are available online at https://www.mdpi.com/1999-4915/11/8/707/s1, Table S1: All partial and complete sequences of classic HAstV *ORF2* gene used in this study. Table S2: Complete sequences of classic HAstV *ORF2* gene used in the study. Table S3: Results of model comparison for evolutionary analysis in this study.
