# Supplementary material for: Molecular Evolution of Classic Human Astrovirus, as Revealed by the Analysis of the Capsid Protein Gene
Source: Viruses. 2019 Aug 1;11(8):707. doi: 10.3390/v11080707 (PMC6722597; doi:10.3390/v11080707)
Supplement: Supplementary file 1 [file viruses-11-00707-s001.zip › Table S3.docx]

**Table S3 Results of model comparison for evolutionary analysis in this study**

| Serotype | Nucleotide substitution model | Clock model | AICM |
| --- | --- | --- | --- |
|  |  |  |  |
|  |  | UCLD | 21825.206 |
| HAstV-1 | TN93+G4 | UCED | 21821.807 |
|  |  | Strict clock | 21063.789 |
|  |  |  |  |
|  |  | UCLD | 12899.068 |
| HAstV-3 | TN93+G4 | UCED | 12753.489 |
|  |  | Strict clock | 12891.746 |
|  |  |  |  |
|  |  | UCLD | 13148.88 |
| HAstV-4 | TN93+I | UCED | 13148.153 |
|  |  | Strict clock | 12937.935 |
|  |  |  |  |
|  |  | UCLD | 10746.102 |
| HAstV-5 | TN93+G4 | UCED | 10744.626 |
|  |  | Strict clock | 10638.041 |
|  |  |  |  |
|  |  | UCLD | 75048.092 |
| All serotypes | GTR+I+G4 | UCED | 74976.21 |
|  |  | Strict clock | 72899.564 |
|  |  |  |  |

UCLD: Relaxed uncorrelated lognormal distribution.

UCED: Relaxed uncorrelated exponential distribution.

AICM: Information Criterion through MCMC, and the lowest AICM value was used.
