# Supplementary material for: Molecular Evolution of Classic Human Astrovirus, as Revealed by the Analysis of the Capsid Protein Gene
Source: Viruses. 2019 Aug 1;11(8):707. doi: 10.3390/v11080707 (PMC6722597; doi:10.3390/v11080707)
Supplement: Supplementary file 1 [file viruses-11-00707-s001.zip › Table S1.docx]

**Table S1 All partial and complete sequences of classic HAstV ORF2 gene used in this study**

| **Serotype** | **Accession Number** | **Country** | **Continent** | **Date** |
| --- | --- | --- | --- | --- |
| HAstV-1 | AY324858 | Argentina | South America | 1995.7-1998.10 |
| HAstV-1 | AY324859 | Argentina | South America | 1995.7-1998.10 |
| HAstV-1 | AY324860 | Argentina | South America | 1995.7-1998.10 |
| HAstV-1 | AY324861 | Argentina | South America | 1995.7-1998.10 |
| HAstV-1 | AY324862 | Argentina | South America | 1995.7-1998.10 |
| HAstV-1 | AY324863 | Argentina | South America | 1995.7-1998.10 |
| HAstV-1 | U49212 | Australia | Oceania | 1995 |
| HAstV-1 | U49213 | Australia | Oceania | 1995 |
| HAstV-1 | U49214 | Australia | Oceania | 1995 |
| HAstV-1 | U49215 | Australia | Oceania | 1995 |
| HAstV-1 | U49216 | Australia | Oceania | 1995 |
| HAstV-1 | U49217 | Australia | Oceania | 1995 |
| HAstV-1 | AF175253 | Australia | Oceania | 1997 |
| HAstV-1 | AF175254 | Australia | Oceania | 1997 |
| HAstV-1 | AF175255 | Australia | Oceania | 1997 |
| HAstV-1 | AF175256 | Australia | Oceania | 1997 |
| HAstV-1 | AF175257 | Australia | Oceania | 1998 |
| HAstV-1 | AF175258 | Australia | Oceania | 1998 |
| HAstV-1 | KC137237 | Australia | Oceania | 2009 |
| HAstV-1 | KC137238 | Australia | Oceania | 2009 |
| HAstV-1 | KC137239 | Australia | Oceania | 2009 |
| HAstV-1 | KC137245 | Australia | Oceania | 2009 |
| HAstV-1 | KC137240 | Australia | Oceania | 2010 |
| HAstV-1 | KC137241 | Australia | Oceania | 2010 |
| HAstV-1 | KC137242 | Australia | Oceania | 2010 |
| HAstV-1 | KC137243 | Australia | Oceania | 2010 |
| HAstV-1 | KC137244 | Australia | Oceania | 2010 |
| HAstV-1 | EU333900 | Bangladesh | Asia | 2005 |
| HAstV-1 | EU333901 | Bangladesh | Asia | 2005 |
| HAstV-1 | EU333902 | Bangladesh | Asia | 2005 |
| HAstV-1 | EU333903 | Bangladesh | Asia | 2005 |
| HAstV-1 | KF420131 | Bangladesh | Asia | 2010 |
| HAstV-1 | KF420132 | Bangladesh | Asia | 2010 |
| HAstV-1 | KF420133 | Bangladesh | Asia | 2010 |
| HAstV-1 | KF420138 | Bangladesh | Asia | 2010 |
| HAstV-1 | KF420143 | Bangladesh | Asia | 2010 |
| HAstV-1 | KF420144 | Bangladesh | Asia | 2010 |
| HAstV-1 | KF420145 | Bangladesh | Asia | 2010 |
| HAstV-1 | KF420146 | Bangladesh | Asia | 2010 |
| HAstV-1 | KF420134 | Bangladesh | Asia | 2011 |
| HAstV-1 | KF420141 | Bangladesh | Asia | 2011 |
| HAstV-1 | KF420147 | Bangladesh | Asia | 2011 |
| HAstV-1 | KF420148 | Bangladesh | Asia | 2011 |
| HAstV-1 | KF420149 | Bangladesh | Asia | 2011 |
| HAstV-1 | KF420130 | Bangladesh | Asia | 2012 |
| HAstV-1 | KF420135 | Bangladesh | Asia | 2012 |
| HAstV-1 | KF420136 | Bangladesh | Asia | 2012 |
| HAstV-1 | KF420139 | Bangladesh | Asia | 2012 |
| HAstV-1 | KF420140 | Bangladesh | Asia | 2012 |
| HAstV-1 | KF420142 | Bangladesh | Asia | 2012 |
| HAstV-1 | EF535737 | Brazil | South America | 1983 |
| HAstV-1 | EF535738 | Brazil | South America | 1983 |
| HAstV-1 | EF535739 | Brazil | South America | 1983 |
| HAstV-1 | EF535740 | Brazil | South America | 1984 |
| HAstV-1 | DQ917375 | Brazil | South America | 1990 |
| HAstV-1 | DQ917377 | Brazil | South America | 1990 |
| HAstV-1 | DQ917381 | Brazil | South America | 1990 |
| HAstV-1 | DQ917388 | Brazil | South America | 1990 |
| HAstV-1 | DQ917376 | Brazil | South America | 1991 |
| HAstV-1 | DQ917378 | Brazil | South America | 1991 |
| HAstV-1 | DQ917379 | Brazil | South America | 1991 |
| HAstV-1 | DQ917380 | Brazil | South America | 1991 |
| HAstV-1 | DQ917383 | Brazil | South America | 1991 |
| HAstV-1 | DQ917384 | Brazil | South America | 1991 |
| HAstV-1 | DQ917385 | Brazil | South America | 1991 |
| HAstV-1 | DQ917386 | Brazil | South America | 1991 |
| HAstV-1 | DQ917387 | Brazil | South America | 1991 |
| HAstV-1 | DQ917390 | Brazil | South America | 1991 |
| HAstV-1 | DQ917389 | Brazil | South America | 1992 |
| HAstV-1 | EF535733 | Brazil | South America | 1993 |
| HAstV-1 | EF535734 | Brazil | South America | 1993 |
| HAstV-1 | EF535735 | Brazil | South America | 1993 |
| HAstV-1 | EF535736 | Brazil | South America | 1993 |
| HAstV-1 | EF535741 | Brazil | South America | 1993 |
| HAstV-1 | EF535742 | Brazil | South America | 1993 |
| HAstV-1 | EF535743 | Brazil | South America | 1993 |
| HAstV-1 | EF535744 | Brazil | South America | 1993 |
| HAstV-1 | EF535745 | Brazil | South America | 1993 |
| HAstV-1 | EF535746 | Brazil | South America | 1993 |
| HAstV-1 | EF535747 | Brazil | South America | 1993 |
| HAstV-1 | EF535748 | Brazil | South America | 1993 |
| HAstV-1 | EF535749 | Brazil | South America | 1993 |
| HAstV-1 | EF535750 | Brazil | South America | 1993 |
| HAstV-1 | EF535751 | Brazil | South America | 1993 |
| HAstV-1 | EF535752 | Brazil | South America | 1993 |
| HAstV-1 | EF535753 | Brazil | South America | 1993 |
| HAstV-1 | EF535754 | Brazil | South America | 1993 |
| HAstV-1 | EF535755 | Brazil | South America | 1993 |
| HAstV-1 | EF535756 | Brazil | South America | 1993 |
| HAstV-1 | EF535757 | Brazil | South America | 1993 |
| HAstV-1 | EF535758 | Brazil | South America | 1993 |
| HAstV-1 | EF535759 | Brazil | South America | 1993 |
| HAstV-1 | EF535760 | Brazil | South America | 1994 |
| HAstV-1 | EF535761 | Brazil | South America | 1994 |
| HAstV-1 | EF535762 | Brazil | South America | 1994 |
| HAstV-1 | EF535763 | Brazil | South America | 1994 |
| HAstV-1 | EF535764 | Brazil | South America | 1994 |
| HAstV-1 | EF535765 | Brazil | South America | 1994 |
| HAstV-1 | EF535766 | Brazil | South America | 1998 |
| HAstV-1 | EF535767 | Brazil | South America | 1998 |
| HAstV-1 | EF535768 | Brazil | South America | 1998 |
| HAstV-1 | EF535769 | Brazil | South America | 1998 |
| HAstV-1 | EF535770 | Brazil | South America | 1998 |
| HAstV-1 | EF535777 | Brazil | South America | 1998 |
| HAstV-1 | EF535778 | Brazil | South America | 1998 |
| HAstV-1 | EF535779 | Brazil | South America | 1998 |
| HAstV-1 | EF535780 | Brazil | South America | 1998 |
| HAstV-1 | EF535781 | Brazil | South America | 1998 |
| HAstV-1 | EF535782 | Brazil | South America | 1998 |
| HAstV-1 | EF535771 | Brazil | South America | 1999 |
| HAstV-1 | EF535772 | Brazil | South America | 1999 |
| HAstV-1 | EF535773 | Brazil | South America | 1999 |
| HAstV-1 | EF535774 | Brazil | South America | 1999 |
| HAstV-1 | EF535775 | Brazil | South America | 1999 |
| HAstV-1 | EF535783 | Brazil | South America | 1999 |
| HAstV-1 | EF535784 | Brazil | South America | 1999 |
| HAstV-1 | EF535785 | Brazil | South America | 1999 |
| HAstV-1 | EF535786 | Brazil | South America | 1999 |
| HAstV-1 | EF535787 | Brazil | South America | 1999 |
| HAstV-1 | EF535788 | Brazil | South America | 1999 |
| HAstV-1 | EF535789 | Brazil | South America | 1999 |
| HAstV-1 | EF535790 | Brazil | South America | 1999 |
| HAstV-1 | EF535791 | Brazil | South America | 1999 |
| HAstV-1 | EF535792 | Brazil | South America | 1999 |
| HAstV-1 | EF535793 | Brazil | South America | 1999 |
| HAstV-1 | DQ071651 | Brazil | South America | 1999 |
| HAstV-1 | DQ071652 | Brazil | South America | 1999 |
| HAstV-1 | DQ071653 | Brazil | South America | 1999 |
| HAstV-1 | DQ071654 | Brazil | South America | 1999 |
| HAstV-1 | DQ071656 | Brazil | South America | 1999 |
| HAstV-1 | EF535776 | Brazil | South America | 2000 |
| HAstV-1 | AY846636 | Brazil | South America | 2000 |
| HAstV-1 | AY846637 | Brazil | South America | 2000 |
| HAstV-1 | AY846638 | Brazil | South America | 2000 |
| HAstV-1 | AY846639 | Brazil | South America | 2000 |
| HAstV-1 | DQ381474 | Brazil | South America | 2004 |
| HAstV-1 | DQ381475 | Brazil | South America | 2004 |
| HAstV-1 | DQ381476 | Brazil | South America | 2004 |
| HAstV-1 | DQ381477 | Brazil | South America | 2004 |
| HAstV-1 | DQ381478 | Brazil | South America | 2004 |
| HAstV-1 | DQ381479 | Brazil | South America | 2004 |
| HAstV-1 | DQ381480 | Brazil | South America | 2004 |
| HAstV-1 | DQ381481 | Brazil | South America | 2004 |
| HAstV-1 | DQ381482 | Brazil | South America | 2004 |
| HAstV-1 | DQ381483 | Brazil | South America | 2004 |
| HAstV-1 | DQ381484 | Brazil | South America | 2004 |
| HAstV-1 | DQ381485 | Brazil | South America | 2004 |
| HAstV-1 | DQ381486 | Brazil | South America | 2004 |
| HAstV-1 | DQ381487 | Brazil | South America | 2004 |
| HAstV-1 | DQ381488 | Brazil | South America | 2004 |
| HAstV-1 | DQ381489 | Brazil | South America | 2004 |
| HAstV-1 | DQ381490 | Brazil | South America | 2004 |
| HAstV-1 | DQ381491 | Brazil | South America | 2004 |
| HAstV-1 | DQ381492 | Brazil | South America | 2004 |
| HAstV-1 | DQ381493 | Brazil | South America | 2004 |
| HAstV-1 | DQ381494 | Brazil | South America | 2004 |
| HAstV-1 | DQ381495 | Brazil | South America | 2004 |
| HAstV-1 | DQ381496 | Brazil | South America | 2004 |
| HAstV-1 | DQ381497 | Brazil | South America | 2004 |
| HAstV-1 | DQ381498 | Brazil | South America | 2004 |
| HAstV-1 | KM269039 | Brazil | South America | 2004 |
| HAstV-1 | EF547371 | Brazil | South America | 2005 |
| HAstV-1 | KM269040 | Brazil | South America | 2005 |
| HAstV-1 | KM269041 | Brazil | South America | 2005 |
| HAstV-1 | KM269042 | Brazil | South America | 2005 |
| HAstV-1 | KM269043 | Brazil | South America | 2005 |
| HAstV-1 | KM269044 | Brazil | South America | 2005 |
| HAstV-1 | KM269045 | Brazil | South America | 2005 |
| HAstV-1 | KM269046 | Brazil | South America | 2005 |
| HAstV-1 | KM269047 | Brazil | South America | 2005 |
| HAstV-1 | KM269048 | Brazil | South America | 2005 |
| HAstV-1 | KM269049 | Brazil | South America | 2005 |
| HAstV-1 | KM269050 | Brazil | South America | 2006 |
| HAstV-1 | KM269051 | Brazil | South America | 2007 |
| HAstV-1 | KM459013 | Brazil | South America | 2008 |
| HAstV-1 | KM269052 | Brazil | South America | 2008 |
| HAstV-1 | JN799266 | Brazil | South America | 2009 |
| HAstV-1 | JN799267 | Brazil | South America | 2009 |
| HAstV-1 | JN799268 | Brazil | South America | 2009 |
| HAstV-1 | JN799269 | Brazil | South America | 2009 |
| HAstV-1 | KM269053 | Brazil | South America | 2009 |
| HAstV-1 | KM269054 | Brazil | South America | 2009 |
| HAstV-1 | KM269057 | Brazil | South America | 2009 |
| HAstV-1 | JN799270 | Brazil | South America | 2010 |
| HAstV-1 | JN799271 | Brazil | South America | 2010 |
| HAstV-1 | KM269055 | Brazil | South America | 2010 |
| HAstV-1 | KM269056 | Brazil | South America | 2010 |
| HAstV-1 | KM269058 | Brazil | South America | 2010 |
| HAstV-1 | KM269059 | Brazil | South America | 2010 |
| HAstV-1 | KP162256 | Bulgaria | Europe | 2009 |
| HAstV-1 | KP162257 | Bulgaria | Europe | 2009 |
| HAstV-1 | KP162258 | Bulgaria | Europe | 2009 |
| HAstV-1 | KP162259 | Bulgaria | Europe | 2009 |
| HAstV-1 | KP162260 | Bulgaria | Europe | 2009 |
| HAstV-1 | KP162261 | Bulgaria | Europe | 2009 |
| HAstV-1 | GU384239 | China | Asia | 2005 |
| HAstV-1 | GU384240 | China | Asia | 2005 |
| HAstV-1 | GU384241 | China | Asia | 2005 |
| HAstV-1 | GU384242 | China | Asia | 2005 |
| HAstV-1 | GU384244 | China | Asia | 2005 |
| HAstV-1 | GU384245 | China | Asia | 2005 |
| HAstV-1 | GU384246 | China | Asia | 2005 |
| HAstV-1 | GU384247 | China | Asia | 2005 |
| HAstV-1 | GU384248 | China | Asia | 2005 |
| HAstV-1 | GU384250 | China | Asia | 2005 |
| HAstV-1 | GU384251 | China | Asia | 2005 |
| HAstV-1 | GU384252 | China | Asia | 2005 |
| HAstV-1 | GU384259 | China | Asia | 2005 |
| HAstV-1 | GQ169027 | China | Asia | 2005 |
| HAstV-1 | GQ169028 | China | Asia | 2005 |
| HAstV-1 | GQ169029 | China | Asia | 2005 |
| HAstV-1 | GQ169030 | China | Asia | 2005 |
| HAstV-1 | GQ169031 | China | Asia | 2005 |
| HAstV-1 | GQ169032 | China | Asia | 2005 |
| HAstV-1 | GQ169033 | China | Asia | 2005 |
| HAstV-1 | GQ169034 | China | Asia | 2005 |
| HAstV-1 | GQ169035 | China | Asia | 2005 |
| HAstV-1 | FJ755364 | China | Asia | 2005 |
| HAstV-1 | FJ755365 | China | Asia | 2005 |
| HAstV-1 | FJ755366 | China | Asia | 2005 |
| HAstV-1 | FJ755367 | China | Asia | 2005 |
| HAstV-1 | FJ755368 | China | Asia | 2005 |
| HAstV-1 | FJ755369 | China | Asia | 2005 |
| HAstV-1 | FJ755370 | China | Asia | 2005 |
| HAstV-1 | FJ755371 | China | Asia | 2005 |
| HAstV-1 | FJ755372 | China | Asia | 2005 |
| HAstV-1 | FJ755373 | China | Asia | 2005 |
| HAstV-1 | FJ755374 | China | Asia | 2005 |
| HAstV-1 | FJ755375 | China | Asia | 2005 |
| HAstV-1 | FJ755376 | China | Asia | 2005 |
| HAstV-1 | FJ755377 | China | Asia | 2005 |
| HAstV-1 | FJ755378 | China | Asia | 2005 |
| HAstV-1 | FJ755379 | China | Asia | 2005 |
| HAstV-1 | FJ755402 | China | Asia | 2005 |
| HAstV-1 | HM212467 | China | Asia | 2006 |
| HAstV-1 | HM212468 | China | Asia | 2006 |
| HAstV-1 | HM212472 | China | Asia | 2006 |
| HAstV-1 | GU384253 | China | Asia | 2006 |
| HAstV-1 | GU384254 | China | Asia | 2006 |
| HAstV-1 | GU384255 | China | Asia | 2006 |
| HAstV-1 | GU384256 | China | Asia | 2006 |
| HAstV-1 | GU384257 | China | Asia | 2006 |
| HAstV-1 | GU384260 | China | Asia | 2006 |
| HAstV-1 | FJ755380 | China | Asia | 2006 |
| HAstV-1 | FJ755381 | China | Asia | 2006 |
| HAstV-1 | FJ755382 | China | Asia | 2006 |
| HAstV-1 | FJ755383 | China | Asia | 2006 |
| HAstV-1 | FJ755384 | China | Asia | 2006 |
| HAstV-1 | FJ755403 | China | Asia | 2006 |
| HAstV-1 | HM212469 | China | Asia | 2007 |
| HAstV-1 | HM212470 | China | Asia | 2007 |
| HAstV-1 | HM212471 | China | Asia | 2007 |
| HAstV-1 | HM212473 | China | Asia | 2007 |
| HAstV-1 | HM212474 | China | Asia | 2007 |
| HAstV-1 | HM212475 | China | Asia | 2007 |
| HAstV-1 | HM212476 | China | Asia | 2007 |
| HAstV-1 | HM212477 | China | Asia | 2007 |
| HAstV-1 | HM212478 | China | Asia | 2007 |
| HAstV-1 | GQ405855 | China | Asia | 2007 |
| HAstV-1 | GQ405856 | China | Asia | 2007 |
| HAstV-1 | GQ323783 | China | Asia | 2007 |
| HAstV-1 | GQ323784 | China | Asia | 2007 |
| HAstV-1 | GQ323785 | China | Asia | 2007 |
| HAstV-1 | GQ323786 | China | Asia | 2007 |
| HAstV-1 | GQ323787 | China | Asia | 2007 |
| HAstV-1 | GQ323788 | China | Asia | 2007 |
| HAstV-1 | GQ323789 | China | Asia | 2007 |
| HAstV-1 | GQ323790 | China | Asia | 2007 |
| HAstV-1 | GQ323791 | China | Asia | 2007 |
| HAstV-1 | GQ323792 | China | Asia | 2007 |
| HAstV-1 | GQ323793 | China | Asia | 2007 |
| HAstV-1 | GQ323794 | China | Asia | 2007 |
| HAstV-1 | GQ323795 | China | Asia | 2007 |
| HAstV-1 | GQ323796 | China | Asia | 2007 |
| HAstV-1 | GQ323797 | China | Asia | 2007 |
| HAstV-1 | GQ323798 | China | Asia | 2007 |
| HAstV-1 | GQ323799 | China | Asia | 2007 |
| HAstV-1 | GQ323800 | China | Asia | 2007 |
| HAstV-1 | GQ323801 | China | Asia | 2007 |
| HAstV-1 | GQ323802 | China | Asia | 2007 |
| HAstV-1 | GQ323803 | China | Asia | 2007 |
| HAstV-1 | GQ323804 | China | Asia | 2007 |
| HAstV-1 | GQ323805 | China | Asia | 2007 |
| HAstV-1 | GQ323806 | China | Asia | 2007 |
| HAstV-1 | FJ755385 | China | Asia | 2007 |
| HAstV-1 | FJ755386 | China | Asia | 2007 |
| HAstV-1 | FJ755387 | China | Asia | 2007 |
| HAstV-1 | FJ755388 | China | Asia | 2007 |
| HAstV-1 | FJ755389 | China | Asia | 2007 |
| HAstV-1 | FJ755391 | China | Asia | 2007 |
| HAstV-1 | FJ755392 | China | Asia | 2007 |
| HAstV-1 | FJ755393 | China | Asia | 2007 |
| HAstV-1 | FJ755394 | China | Asia | 2007 |
| HAstV-1 | FJ755395 | China | Asia | 2007 |
| HAstV-1 | FJ755396 | China | Asia | 2007 |
| HAstV-1 | FJ755397 | China | Asia | 2007 |
| HAstV-1 | FJ755398 | China | Asia | 2007 |
| HAstV-1 | FJ755399 | China | Asia | 2007 |
| HAstV-1 | FJ755400 | China | Asia | 2007 |
| HAstV-1 | FJ755401 | China | Asia | 2007 |
| HAstV-1 | FJ755404 | China | Asia | 2007 |
| HAstV-1 | FJ755405 | China | Asia | 2007 |
| HAstV-1 | FJ792842 | China | Asia | 2008 |
| HAstV-1 | FJ538930 | China | Asia | 2008 |
| HAstV-1 | FJ538931 | China | Asia | 2008 |
| HAstV-1 | FJ538932 | China | Asia | 2008 |
| HAstV-1 | FJ538933 | China | Asia | 2008 |
| HAstV-1 | FJ538934 | China | Asia | 2008 |
| HAstV-1 | FJ538935 | China | Asia | 2008 |
| HAstV-1 | FJ538936 | China | Asia | 2008 |
| HAstV-1 | FJ538937 | China | Asia | 2008 |
| HAstV-1 | FJ538938 | China | Asia | 2008 |
| HAstV-1 | FJ538939 | China | Asia | 2008 |
| HAstV-1 | FJ538940 | China | Asia | 2008 |
| HAstV-1 | FJ538941 | China | Asia | 2008 |
| HAstV-1 | FJ538942 | China | Asia | 2008 |
| HAstV-1 | FJ538943 | China | Asia | 2008 |
| HAstV-1 | FJ538944 | China | Asia | 2008 |
| HAstV-1 | FJ538945 | China | Asia | 2008 |
| HAstV-1 | FJ538946 | China | Asia | 2008 |
| HAstV-1 | HM450036 | China | Asia | 2008 |
| HAstV-1 | HM450037 | China | Asia | 2008 |
| HAstV-1 | HM450038 | China | Asia | 2008 |
| HAstV-1 | HM450039 | China | Asia | 2008 |
| HAstV-1 | HM450040 | China | Asia | 2008 |
| HAstV-1 | HM450041 | China | Asia | 2008 |
| HAstV-1 | HM450042 | China | Asia | 2008 |
| HAstV-1 | HM450043 | China | Asia | 2008 |
| HAstV-1 | GQ323807 | China | Asia | 2008 |
| HAstV-1 | GQ323808 | China | Asia | 2008 |
| HAstV-1 | GQ323809 | China | Asia | 2008 |
| HAstV-1 | GQ323810 | China | Asia | 2008 |
| HAstV-1 | GQ323811 | China | Asia | 2008 |
| HAstV-1 | GQ323812 | China | Asia | 2008 |
| HAstV-1 | GQ323813 | China | Asia | 2008 |
| HAstV-1 | GQ323814 | China | Asia | 2008 |
| HAstV-1 | GQ323815 | China | Asia | 2008 |
| HAstV-1 | GQ323816 | China | Asia | 2008 |
| HAstV-1 | GQ323817 | China | Asia | 2008 |
| HAstV-1 | GQ323818 | China | Asia | 2008 |
| HAstV-1 | GQ323819 | China | Asia | 2008 |
| HAstV-1 | GQ323820 | China | Asia | 2008 |
| HAstV-1 | GQ323821 | China | Asia | 2008 |
| HAstV-1 | GQ323822 | China | Asia | 2008 |
| HAstV-1 | GQ323823 | China | Asia | 2008 |
| HAstV-1 | GQ323824 | China | Asia | 2008 |
| HAstV-1 | GQ323825 | China | Asia | 2008 |
| HAstV-1 | GQ323826 | China | Asia | 2008 |
| HAstV-1 | GQ323827 | China | Asia | 2008 |
| HAstV-1 | GQ323828 | China | Asia | 2008 |
| HAstV-1 | GQ323829 | China | Asia | 2008 |
| HAstV-1 | GQ323830 | China | Asia | 2008 |
| HAstV-1 | GQ323831 | China | Asia | 2008 |
| HAstV-1 | GQ323832 | China | Asia | 2008 |
| HAstV-1 | GQ323833 | China | Asia | 2008 |
| HAstV-1 | JF343966 | China | Asia | 2009 |
| HAstV-1 | JF343967 | China | Asia | 2009 |
| HAstV-1 | JF343968 | China | Asia | 2009 |
| HAstV-1 | JF343969 | China | Asia | 2009 |
| HAstV-1 | JF343970 | China | Asia | 2009 |
| HAstV-1 | JF343971 | China | Asia | 2009 |
| HAstV-1 | JF343972 | China | Asia | 2009 |
| HAstV-1 | JF343973 | China | Asia | 2009 |
| HAstV-1 | JF343974 | China | Asia | 2009 |
| HAstV-1 | JF343975 | China | Asia | 2009 |
| HAstV-1 | JF343977 | China | Asia | 2009 |
| HAstV-1 | HM120876 | China | Asia | 2009 |
| HAstV-1 | HM120877 | China | Asia | 2009 |
| HAstV-1 | HM120878 | China | Asia | 2009 |
| HAstV-1 | KF211460 | China | Asia | 2010 |
| HAstV-1 | KF211461 | China | Asia | 2010 |
| HAstV-1 | KF211462 | China | Asia | 2010 |
| HAstV-1 | KF211463 | China | Asia | 2010 |
| HAstV-1 | KF211464 | China | Asia | 2010 |
| HAstV-1 | KF211465 | China | Asia | 2010 |
| HAstV-1 | KF211466 | China | Asia | 2010 |
| HAstV-1 | KF211467 | China | Asia | 2010 |
| HAstV-1 | KF211468 | China | Asia | 2010 |
| HAstV-1 | KF211469 | China | Asia | 2010 |
| HAstV-1 | KF211470 | China | Asia | 2010 |
| HAstV-1 | KF211471 | China | Asia | 2010 |
| HAstV-1 | KF211472 | China | Asia | 2010 |
| HAstV-1 | KF211473 | China | Asia | 2010 |
| HAstV-1 | KF211474 | China | Asia | 2010 |
| HAstV-1 | KF211475 | China | Asia | 2010 |
| HAstV-1 | KF512009 | China | Asia | 2010 |
| HAstV-1 | KP942590 | China | Asia | 2013 |
| HAstV-1 | KP942591 | China | Asia | 2013 |
| HAstV-1 | KP942592 | China | Asia | 2013 |
| HAstV-1 | KP942593 | China | Asia | 2013 |
| HAstV-1 | KJ935470 | China | Asia | 2013 |
| HAstV-1 | KP942582 | China | Asia | 2014 |
| HAstV-1 | KP942583 | China | Asia | 2014 |
| HAstV-1 | KP942584 | China | Asia | 2014 |
| HAstV-1 | KP942585 | China | Asia | 2014 |
| HAstV-1 | KP942586 | China | Asia | 2014 |
| HAstV-1 | KP942587 | China | Asia | 2014 |
| HAstV-1 | KP942588 | China | Asia | 2014 |
| HAstV-1 | KP942589 | China | Asia | 2014 |
| HAstV-1 | KY311969 | China | Asia | 2015 |
| HAstV-1 | KY311970 | China | Asia | 2015 |
| HAstV-1 | KY311971 | China | Asia | 2015 |
| HAstV-1 | KY311972 | China | Asia | 2015 |
| HAstV-1 | KY311973 | China | Asia | 2015 |
| HAstV-1 | KY311974 | China | Asia | 2015 |
| HAstV-1 | KY311975 | China | Asia | 2015 |
| HAstV-1 | KY311976 | China | Asia | 2015 |
| HAstV-1 | KY311977 | China | Asia | 2015 |
| HAstV-1 | KY311978 | China | Asia | 2015 |
| HAstV-1 | KY311979 | China | Asia | 2015 |
| HAstV-1 | KY311980 | China | Asia | 2015 |
| HAstV-1 | KY311981 | China | Asia | 2015 |
| HAstV-1 | KY311982 | China | Asia | 2015 |
| HAstV-1 | KY311983 | China | Asia | 2015 |
| HAstV-1 | KY311984 | China | Asia | 2015 |
| HAstV-1 | KY311985 | China | Asia | 2015 |
| HAstV-1 | KY311986 | China | Asia | 2015 |
| HAstV-1 | KY311987 | China | Asia | 2015 |
| HAstV-1 | KY311988 | China | Asia | 2015 |
| HAstV-1 | KY311989 | China | Asia | 2015 |
| HAstV-1 | KY311990 | China | Asia | 2015 |
| HAstV-1 | KY311991 | China | Asia | 2015 |
| HAstV-1 | KY311993 | China | Asia | 2015 |
| HAstV-1 | KY311994 | China | Asia | 2015 |
| HAstV-1 | KY311995 | China | Asia | 2015 |
| HAstV-1 | KY311996 | China | Asia | 2015 |
| HAstV-1 | KY311997 | China | Asia | 2015 |
| HAstV-1 | KY311998 | China | Asia | 2015 |
| HAstV-1 | KY311999 | China | Asia | 2015 |
| HAstV-1 | KY312000 | China | Asia | 2015 |
| HAstV-1 | KY828141 | China | Asia | 2015 |
| HAstV-1 | KY828140 | China | Asia | 2016 |
| HAstV-1 | MH643741 | China | Asia | 2017 |
| HAstV-1 | AF211962 | Colombia | South America | 1997.06-1999.02 |
| HAstV-1 | AF211963 | Colombia | South America | 1997.06-1999.03 |
| HAstV-1 | AF211964 | Colombia | South America | 1997.06-1999.04 |
| HAstV-1 | AF211965 | Colombia | South America | 1997.06-1999.05 |
| HAstV-1 | HQ674634 | Egypt | Africa | 2007 |
| HAstV-1 | HQ674635 | Egypt | Africa | 2007 |
| HAstV-1 | HQ674636 | Egypt | Africa | 2007 |
| HAstV-1 | HQ674637 | Egypt | Africa | 2007 |
| HAstV-1 | HQ674638 | Egypt | Africa | 2007 |
| HAstV-1 | HQ674639 | Egypt | Africa | 2007 |
| HAstV-1 | HQ674640 | Egypt | Africa | 2007 |
| HAstV-1 | HQ674641 | Egypt | Africa | 2007 |
| HAstV-1 | HQ674642 | Egypt | Africa | 2007 |
| HAstV-1 | AY720892 | Germany | Europe | 2004 |
| HAstV-1 | AY007577 | Germany | Europe | 1997 |
| HAstV-1 | AY007579 | Germany | Europe | 1998 |
| HAstV-1 | AY007580 | Germany | Europe | 1998 |
| HAstV-1 | AY007581 | Germany | Europe | 1998 |
| HAstV-1 | AY007582 | Germany | Europe | 1998 |
| HAstV-1 | AY007583 | Germany | Europe | 1998 |
| HAstV-1 | AY007578 | Germany | Europe | 1999 |
| HAstV-1 | AF395733 | Hungary | Europe | 1995.03-1999.02 |
| HAstV-1 | AF395734 | Hungary | Europe | 1995.03-1999.02 |
| HAstV-1 | AJ620751 | Hungary | Europe | 2002 |
| HAstV-1 | AJ620752 | Hungary | Europe | 2002 |
| HAstV-1 | AJ620755 | Hungary | Europe | 2002 |
| HAstV-1 | AJ620756 | Hungary | Europe | 2002 |
| HAstV-1 | AJ620759 | Hungary | Europe | 2002 |
| HAstV-1 | HQ398856 | Hungary | Europe | 2010 |
| HAstV-1 | KY815035 | India | Asia | 2009 |
| HAstV-1 | AB548400 | India | Asia | 2009 |
| HAstV-1 | AB548401 | India | Asia | 2009 |
| HAstV-1 | AB548402 | India | Asia | 2009 |
| HAstV-1 | AB548403 | India | Asia | 2009 |
| HAstV-1 | AB548405 | India | Asia | 2009 |
| HAstV-1 | AB551373 | India | Asia | 2009 |
| HAstV-1 | AB551374 | India | Asia | 2009 |
| HAstV-1 | AB551375 | India | Asia | 2009 |
| HAstV-1 | AB551376 | India | Asia | 2009 |
| HAstV-1 | AB551377 | India | Asia | 2009 |
| HAstV-1 | AB551379 | India | Asia | 2009 |
| HAstV-1 | AB551380 | India | Asia | 2009 |
| HAstV-1 | KY815036 | India | Asia | 2014 |
| HAstV-1 | KY815037 | India | Asia | 2015 |
| HAstV-1 | KY744138 | Italy | Europe | 1999 |
| HAstV-1 | KY744137 | Italy | Europe | 2000 |
| HAstV-1 | GU216589 | Italy | Europe | 2003 |
| HAstV-1 | GU216590 | Italy | Europe | 2003 |
| HAstV-1 | GU216591 | Italy | Europe | 2003 |
| HAstV-1 | GU216592 | Italy | Europe | 2003 |
| HAstV-1 | KY744139 | Italy | Europe | 2004 |
| HAstV-1 | GU216593 | Italy | Europe | 2004 |
| HAstV-1 | GU216594 | Italy | Europe | 2004 |
| HAstV-1 | GU216595 | Italy | Europe | 2004 |
| HAstV-1 | GU216596 | Italy | Europe | 2004 |
| HAstV-1 | KU508614 | Italy | Europe | 2004 |
| HAstV-1 | JX087965 | Italy | Europe | 2005 |
| HAstV-1 | GU216597 | Italy | Europe | 2005 |
| HAstV-1 | KU508615 | Italy | Europe | 2006 |
| HAstV-1 | KU508616 | Italy | Europe | 2006 |
| HAstV-1 | KU508607 | Italy | Europe | 2007 |
| HAstV-1 | KU508612 | Italy | Europe | 2007 |
| HAstV-1 | KU508613 | Italy | Europe | 2007 |
| HAstV-1 | KY744140 | Italy | Europe | 2008 |
| HAstV-1 | JQ434362 | Italy | Europe | 2008 |
| HAstV-1 | JQ434363 | Italy | Europe | 2008 |
| HAstV-1 | JQ434364 | Italy | Europe | 2008 |
| HAstV-1 | JQ434371 | Italy | Europe | 2008 |
| HAstV-1 | JQ434372 | Italy | Europe | 2008 |
| HAstV-1 | JQ434373 | Italy | Europe | 2008 |
| HAstV-1 | JQ434374 | Italy | Europe | 2008 |
| HAstV-1 | JQ434380 | Italy | Europe | 2008 |
| HAstV-1 | JQ434381 | Italy | Europe | 2008 |
| HAstV-1 | JQ434382 | Italy | Europe | 2008 |
| HAstV-1 | JQ434383 | Italy | Europe | 2008 |
| HAstV-1 | JQ434384 | Italy | Europe | 2008 |
| HAstV-1 | JQ434385 | Italy | Europe | 2008 |
| HAstV-1 | JQ434386 | Italy | Europe | 2008 |
| HAstV-1 | JQ434387 | Italy | Europe | 2008 |
| HAstV-1 | JQ434388 | Italy | Europe | 2008 |
| HAstV-1 | JQ434389 | Italy | Europe | 2008 |
| HAstV-1 | JQ434390 | Italy | Europe | 2008 |
| HAstV-1 | JQ434391 | Italy | Europe | 2008 |
| HAstV-1 | JQ434392 | Italy | Europe | 2008 |
| HAstV-1 | JQ434393 | Italy | Europe | 2008 |
| HAstV-1 | KU508608 | Italy | Europe | 2008 |
| HAstV-1 | JQ434365 | Italy | Europe | 2009 |
| HAstV-1 | JQ434366 | Italy | Europe | 2009 |
| HAstV-1 | JQ434367 | Italy | Europe | 2009 |
| HAstV-1 | JQ434368 | Italy | Europe | 2009 |
| HAstV-1 | JQ434369 | Italy | Europe | 2009 |
| HAstV-1 | JQ434370 | Italy | Europe | 2009 |
| HAstV-1 | JQ434375 | Italy | Europe | 2009 |
| HAstV-1 | JQ434376 | Italy | Europe | 2009 |
| HAstV-1 | JQ434377 | Italy | Europe | 2009 |
| HAstV-1 | JQ434378 | Italy | Europe | 2009 |
| HAstV-1 | JQ434379 | Italy | Europe | 2009 |
| HAstV-1 | KU508609 | Italy | Europe | 2010 |
| HAstV-1 | KY744141 | Italy | Europe | 2011 |
| HAstV-1 | KU508610 | Italy | Europe | 2011 |
| HAstV-1 | KU508611 | Italy | Europe | 2014 |
| HAstV-1 | MK041036 | Italy | Europe | 2017 |
| HAstV-1 | MK041037 | Italy | Europe | 2017 |
| HAstV-1 | MK041038 | Italy | Europe | 2017 |
| HAstV-1 | AB009984 | Japan | Asia | 1992 |
| HAstV-1 | AB000284 | Japan | Asia | 1992 |
| HAstV-1 | AB000287 | Japan | Asia | 1992 |
| HAstV-1 | AB009985 | Japan | Asia | 1993 |
| HAstV-1 | AB000286 | Japan | Asia | 1993 |
| HAstV-1 | AB000292 | Japan | Asia | 1993 |
| HAstV-1 | AB000293 | Japan | Asia | 1993 |
| HAstV-1 | AB000294 | Japan | Asia | 1993 |
| HAstV-1 | AB000295 | Japan | Asia | 1993 |
| HAstV-1 | AB000298 | Japan | Asia | 1993 |
| HAstV-1 | AB000299 | Japan | Asia | 1997 |
| HAstV-1 | AB000300 | Japan | Asia | 1997 |
| HAstV-1 | LC009663 | Japan | Asia | 2007 |
| HAstV-1 | LC009664 | Japan | Asia | 2007 |
| HAstV-1 | LC009662 | Japan | Asia | 2008 |
| HAstV-1 | HM212532 | Japan | Asia | 2008 |
| HAstV-1 | HM212537 | Japan | Asia | 2008 |
| HAstV-1 | HM212533 | Japan | Asia | 2009 |
| HAstV-1 | HM212534 | Japan | Asia | 2009 |
| HAstV-1 | HM212535 | Japan | Asia | 2009 |
| HAstV-1 | HM212536 | Japan | Asia | 2009 |
| HAstV-1 | HM212538 | Japan | Asia | 2009 |
| HAstV-1 | HM212539 | Japan | Asia | 2009 |
| HAstV-1 | HM212540 | Japan | Asia | 2009 |
| HAstV-1 | HM212541 | Japan | Asia | 2009 |
| HAstV-1 | FJ823057 | Japan | Asia | 2006-2007 |
| HAstV-1 | FJ823058 | Japan | Asia | 2006-2007 |
| HAstV-1 | FJ823059 | Japan | Asia | 2006-2007 |
| HAstV-1 | FJ823060 | Japan | Asia | 2006-2007 |
| HAstV-1 | FJ823061 | Japan | Asia | 2006-2007 |
| HAstV-1 | FJ823062 | Japan | Asia | 2006-2007 |
| HAstV-1 | FJ823063 | Japan | Asia | 2006-2007 |
| HAstV-1 | FJ823064 | Japan | Asia | 2006-2007 |
| HAstV-1 | FJ823065 | Japan | Asia | 2006-2007 |
| HAstV-1 | FJ823066 | Japan | Asia | 2006-2007 |
| HAstV-1 | FJ823067 | Japan | Asia | 2006-2007 |
| HAstV-1 | FJ823068 | Japan | Asia | 2006-2007 |
| HAstV-1 | FJ823069 | Japan | Asia | 2006-2007 |
| HAstV-1 | FJ823070 | Japan | Asia | 2006-2007 |
| HAstV-1 | FJ823071 | Japan | Asia | 2006-2007 |
| HAstV-1 | LC459977 | Japan | Asia | 2015 |
| HAstV-1 | LC459980 | Japan | Asia | 2015 |
| HAstV-1 | LC459981 | Japan | Asia | 2015 |
| HAstV-1 | LC459982 | Japan | Asia | 2015 |
| HAstV-1 | LC459983 | Japan | Asia | 2015 |
| HAstV-1 | LC459984 | Japan | Asia | 2015 |
| HAstV-1 | LC459985 | Japan | Asia | 2015 |
| HAstV-1 | LC459978 | Japan | Asia | 2016 |
| HAstV-1 | LC459979 | Japan | Asia | 2016 |
| HAstV-1 | LC459986 | Japan | Asia | 2016 |
| HAstV-1 | LC459987 | Japan | Asia | 2016 |
| HAstV-1 | LC459988 | Japan | Asia | 2016 |
| HAstV-1 | DQ659100 | Korea | Asia | 2005 |
| HAstV-1 | JN887820 | Korea | Asia | 2011 |
| HAstV-1 | KY905156 | Korea | Asia | 2014 |
| HAstV-1 | MK472054 | Korea | Asia | 2016 |
| HAstV-1 | MK430063 | Korea | Asia | 2017 |
| HAstV-1 | KY200610 | Morocco | Africa | 2011 |
| HAstV-1 | AB000290 | Norway | Europe | 1993 |
| HAstV-1 | AB000285 | Pakistan | Asia | 1991 |
| HAstV-1 | AB000301 | Pakistan | Asia | 1993 |
| HAstV-1 | GU247899 | Russia | Europe | 2003 |
| HAstV-1 | GU247900 | Russia | Europe | 2003 |
| HAstV-1 | JF491365 | Russia | Europe | 2004 |
| HAstV-1 | JF491367 | Russia | Europe | 2004 |
| HAstV-1 | JF491368 | Russia | Europe | 2004 |
| HAstV-1 | JF491369 | Russia | Europe | 2004 |
| HAstV-1 | JF491370 | Russia | Europe | 2004 |
| HAstV-1 | JF491371 | Russia | Europe | 2004 |
| HAstV-1 | JF491372 | Russia | Europe | 2004 |
| HAstV-1 | JF491373 | Russia | Europe | 2004 |
| HAstV-1 | JF491374 | Russia | Europe | 2004 |
| HAstV-1 | JF491375 | Russia | Europe | 2004 |
| HAstV-1 | JF491376 | Russia | Europe | 2004 |
| HAstV-1 | JF491377 | Russia | Europe | 2005 |
| HAstV-1 | JF491378 | Russia | Europe | 2005 |
| HAstV-1 | JF491379 | Russia | Europe | 2005 |
| HAstV-1 | JF491381 | Russia | Europe | 2005 |
| HAstV-1 | JF491383 | Russia | Europe | 2005 |
| HAstV-1 | JF491384 | Russia | Europe | 2005 |
| HAstV-1 | JF491385 | Russia | Europe | 2005 |
| HAstV-1 | JF491386 | Russia | Europe | 2005 |
| HAstV-1 | JF491387 | Russia | Europe | 2005 |
| HAstV-1 | JF491388 | Russia | Europe | 2005 |
| HAstV-1 | JF491389 | Russia | Europe | 2005 |
| HAstV-1 | JF491390 | Russia | Europe | 2005 |
| HAstV-1 | GU212644 | Russia | Europe | 2005 |
| HAstV-1 | JF491391 | Russia | Europe | 2006 |
| HAstV-1 | JF491392 | Russia | Europe | 2006 |
| HAstV-1 | JF491393 | Russia | Europe | 2006 |
| HAstV-1 | JF491394 | Russia | Europe | 2006 |
| HAstV-1 | JF491395 | Russia | Europe | 2006 |
| HAstV-1 | JF491396 | Russia | Europe | 2006 |
| HAstV-1 | JF491397 | Russia | Europe | 2006 |
| HAstV-1 | JF491398 | Russia | Europe | 2006 |
| HAstV-1 | JF491400 | Russia | Europe | 2006 |
| HAstV-1 | GU212648 | Russia | Europe | 2006 |
| HAstV-1 | GU212649 | Russia | Europe | 2006 |
| HAstV-1 | JF929181 | Russia | Europe | 2007 |
| HAstV-1 | JF929182 | Russia | Europe | 2007 |
| HAstV-1 | JF929183 | Russia | Europe | 2007 |
| HAstV-1 | GQ373004 | Russia | Europe | 2007 |
| HAstV-1 | GQ373005 | Russia | Europe | 2007 |
| HAstV-1 | GQ373006 | Russia | Europe | 2007 |
| HAstV-1 | GQ373007 | Russia | Europe | 2007 |
| HAstV-1 | GQ373008 | Russia | Europe | 2007 |
| HAstV-1 | GQ373013 | Russia | Europe | 2007 |
| HAstV-1 | FJ866761 | Russia | Europe | 2007 |
| HAstV-1 | GQ351328 | Russia | Europe | 2007 |
| HAstV-1 | JF929184 | Russia | Europe | 2008 |
| HAstV-1 | GU223908 | Russia | Europe | 2008 |
| HAstV-1 | GU223909 | Russia | Europe | 2008 |
| HAstV-1 | GU223910 | Russia | Europe | 2008 |
| HAstV-1 | GU223911 | Russia | Europe | 2008 |
| HAstV-1 | GU223912 | Russia | Europe | 2008 |
| HAstV-1 | GU223913 | Russia | Europe | 2008 |
| HAstV-1 | GU212651 | Russia | Europe | 2008 |
| HAstV-1 | HM630260 | Russia | Europe | 2009 |
| HAstV-1 | HM630261 | Russia | Europe | 2009 |
| HAstV-1 | HM630262 | Russia | Europe | 2009 |
| HAstV-1 | HM630263 | Russia | Europe | 2009 |
| HAstV-1 | HM630264 | Russia | Europe | 2009 |
| HAstV-1 | HM630265 | Russia | Europe | 2009 |
| HAstV-1 | HM630266 | Russia | Europe | 2009 |
| HAstV-1 | HM630267 | Russia | Europe | 2009 |
| HAstV-1 | HM630268 | Russia | Europe | 2009 |
| HAstV-1 | HM630269 | Russia | Europe | 2009 |
| HAstV-1 | HM630270 | Russia | Europe | 2009 |
| HAstV-1 | HM630271 | Russia | Europe | 2009 |
| HAstV-1 | HM630272 | Russia | Europe | 2009 |
| HAstV-1 | HM630273 | Russia | Europe | 2009 |
| HAstV-1 | JF929185 | Russia | Europe | 2010 |
| HAstV-1 | JF929186 | Russia | Europe | 2010 |
| HAstV-1 | JF929187 | Russia | Europe | 2010 |
| HAstV-1 | JF929188 | Russia | Europe | 2010 |
| HAstV-1 | JF929189 | Russia | Europe | 2010 |
| HAstV-1 | JF929190 | Russia | Europe | 2010 |
| HAstV-1 | JF929191 | Russia | Europe | 2010 |
| HAstV-1 | JF929192 | Russia | Europe | 2010 |
| HAstV-1 | JF491402 | Russia | Europe | 2010 |
| HAstV-1 | JF491404 | Russia | Europe | 2010 |
| HAstV-1 | JF491405 | Russia | Europe | 2010 |
| HAstV-1 | JF491406 | Russia | Europe | 2010 |
| HAstV-1 | JF491407 | Russia | Europe | 2010 |
| HAstV-1 | JF491408 | Russia | Europe | 2010 |
| HAstV-1 | JF491409 | Russia | Europe | 2010 |
| HAstV-1 | JF491410 | Russia | Europe | 2010 |
| HAstV-1 | JF491411 | Russia | Europe | 2010 |
| HAstV-1 | JF491412 | Russia | Europe | 2010 |
| HAstV-1 | JF491413 | Russia | Europe | 2010 |
| HAstV-1 | JF491414 | Russia | Europe | 2010 |
| HAstV-1 | JF491415 | Russia | Europe | 2010 |
| HAstV-1 | JF491416 | Russia | Europe | 2010 |
| HAstV-1 | JF491417 | Russia | Europe | 2010 |
| HAstV-1 | JF491418 | Russia | Europe | 2010 |
| HAstV-1 | JF491419 | Russia | Europe | 2010 |
| HAstV-1 | JF491421 | Russia | Europe | 2010 |
| HAstV-1 | JF491422 | Russia | Europe | 2010 |
| HAstV-1 | JF491423 | Russia | Europe | 2010 |
| HAstV-1 | JF491424 | Russia | Europe | 2010 |
| HAstV-1 | JF491425 | Russia | Europe | 2010 |
| HAstV-1 | JF491426 | Russia | Europe | 2010 |
| HAstV-1 | JF491427 | Russia | Europe | 2010 |
| HAstV-1 | JF491428 | Russia | Europe | 2010 |
| HAstV-1 | JF491429 | Russia | Europe | 2010 |
| HAstV-1 | MH318020 | Russia | Europe | 2010 |
| HAstV-1 | MG932557 | Russia | Europe | 2010 |
| HAstV-1 | MG932558 | Russia | Europe | 2010 |
| HAstV-1 | MG932559 | Russia | Europe | 2010 |
| HAstV-1 | MG932560 | Russia | Europe | 2010 |
| HAstV-1 | MG932561 | Russia | Europe | 2010 |
| HAstV-1 | MG932562 | Russia | Europe | 2010 |
| HAstV-1 | MG932563 | Russia | Europe | 2010 |
| HAstV-1 | MG932564 | Russia | Europe | 2010 |
| HAstV-1 | MG932565 | Russia | Europe | 2010 |
| HAstV-1 | MG932566 | Russia | Europe | 2010 |
| HAstV-1 | MG932567 | Russia | Europe | 2010 |
| HAstV-1 | KC285125 | Russia | Europe | 2010 |
| HAstV-1 | KC285126 | Russia | Europe | 2010 |
| HAstV-1 | KC285127 | Russia | Europe | 2010 |
| HAstV-1 | KC285124 | Russia | Europe | 2010 |
| HAstV-1 | JF491403 | Russia | Europe | 2010 |
| HAstV-1 | KM923911 | Russia | Europe | 2011 |
| HAstV-1 | JN203050 | Russia | Europe | 2011 |
| HAstV-1 | JN203051 | Russia | Europe | 2011 |
| HAstV-1 | MH318026 | Russia | Europe | 2011 |
| HAstV-1 | MH318028 | Russia | Europe | 2011 |
| HAstV-1 | KC285128 | Russia | Europe | 2011 |
| HAstV-1 | KC285129 | Russia | Europe | 2011 |
| HAstV-1 | KC285130 | Russia | Europe | 2011 |
| HAstV-1 | KC285131 | Russia | Europe | 2011 |
| HAstV-1 | KC285132 | Russia | Europe | 2011 |
| HAstV-1 | KC285133 | Russia | Europe | 2011 |
| HAstV-1 | KC285134 | Russia | Europe | 2011 |
| HAstV-1 | KC285135 | Russia | Europe | 2011 |
| HAstV-1 | KC285136 | Russia | Europe | 2011 |
| HAstV-1 | KC285137 | Russia | Europe | 2011 |
| HAstV-1 | KC285138 | Russia | Europe | 2011 |
| HAstV-1 | KC285139 | Russia | Europe | 2011 |
| HAstV-1 | KC285140 | Russia | Europe | 2011 |
| HAstV-1 | KC285141 | Russia | Europe | 2011 |
| HAstV-1 | KC285142 | Russia | Europe | 2011 |
| HAstV-1 | KC285143 | Russia | Europe | 2011 |
| HAstV-1 | KC285144 | Russia | Europe | 2011 |
| HAstV-1 | KC285145 | Russia | Europe | 2011 |
| HAstV-1 | KC285146 | Russia | Europe | 2011 |
| HAstV-1 | KC285147 | Russia | Europe | 2011 |
| HAstV-1 | KC285148 | Russia | Europe | 2011 |
| HAstV-1 | KC285149 | Russia | Europe | 2011 |
| HAstV-1 | KC285150 | Russia | Europe | 2011 |
| HAstV-1 | KC285151 | Russia | Europe | 2011 |
| HAstV-1 | KC285153 | Russia | Europe | 2011 |
| HAstV-1 | KC285154 | Russia | Europe | 2011 |
| HAstV-1 | KC285155 | Russia | Europe | 2011 |
| HAstV-1 | KC285156 | Russia | Europe | 2011 |
| HAstV-1 | KC285157 | Russia | Europe | 2011 |
| HAstV-1 | KC285158 | Russia | Europe | 2011 |
| HAstV-1 | KC285159 | Russia | Europe | 2011 |
| HAstV-1 | KC285161 | Russia | Europe | 2011 |
| HAstV-1 | KC285162 | Russia | Europe | 2011 |
| HAstV-1 | KC285163 | Russia | Europe | 2011 |
| HAstV-1 | KC285164 | Russia | Europe | 2011 |
| HAstV-1 | KC285165 | Russia | Europe | 2011 |
| HAstV-1 | KC285166 | Russia | Europe | 2011 |
| HAstV-1 | KC285167 | Russia | Europe | 2011 |
| HAstV-1 | KC285168 | Russia | Europe | 2011 |
| HAstV-1 | KC285169 | Russia | Europe | 2011 |
| HAstV-1 | KC285170 | Russia | Europe | 2011 |
| HAstV-1 | KC285171 | Russia | Europe | 2011 |
| HAstV-1 | KF039914 | Russia | Europe | 2011 |
| HAstV-1 | KF039915 | Russia | Europe | 2011 |
| HAstV-1 | KF039916 | Russia | Europe | 2011 |
| HAstV-1 | KM923912 | Russia | Europe | 2012 |
| HAstV-1 | KM923913 | Russia | Europe | 2012 |
| HAstV-1 | MG932568 | Russia | Europe | 2012 |
| HAstV-1 | KC285172 | Russia | Europe | 2012 |
| HAstV-1 | KC285173 | Russia | Europe | 2012 |
| HAstV-1 | KC285174 | Russia | Europe | 2012 |
| HAstV-1 | KC285175 | Russia | Europe | 2012 |
| HAstV-1 | KC285176 | Russia | Europe | 2012 |
| HAstV-1 | KC285179 | Russia | Europe | 2012 |
| HAstV-1 | KC285180 | Russia | Europe | 2012 |
| HAstV-1 | KC285182 | Russia | Europe | 2012 |
| HAstV-1 | KC285183 | Russia | Europe | 2012 |
| HAstV-1 | KC285184 | Russia | Europe | 2012 |
| HAstV-1 | KC285185 | Russia | Europe | 2012 |
| HAstV-1 | KC285186 | Russia | Europe | 2012 |
| HAstV-1 | KC285188 | Russia | Europe | 2012 |
| HAstV-1 | KF039917 | Russia | Europe | 2012 |
| HAstV-1 | KF039919 | Russia | Europe | 2012 |
| HAstV-1 | KF039921 | Russia | Europe | 2012 |
| HAstV-1 | KT749767 | Russia | Europe | 2013 |
| HAstV-1 | KP208780 | Russia | Europe | 2013 |
| HAstV-1 | KT749768 | Russia | Europe | 2014 |
| HAstV-1 | KT749769 | Russia | Europe | 2014 |
| HAstV-1 | KT749770 | Russia | Europe | 2014 |
| HAstV-1 | KT749771 | Russia | Europe | 2015 |
| HAstV-1 | KT749772 | Russia | Europe | 2015 |
| HAstV-1 | KT749773 | Russia | Europe | 2015 |
| HAstV-1 | KT749774 | Russia | Europe | 2015 |
| HAstV-1 | MG932577 | Russia | Europe | 2016 |
| HAstV-1 | MG932578 | Russia | Europe | 2016 |
| HAstV-1 | MG932582 | Russia | Europe | 2017 |
| HAstV-1 | MG932583 | Russia | Europe | 2017 |
| HAstV-1 | MG932585 | Russia | Europe | 2017 |
| HAstV-1 | MG932588 | Russia | Europe | 2017 |
| HAstV-1 | MG932589 | Russia | Europe | 2017 |
| HAstV-1 | MG932590 | Russia | Europe | 2017 |
| HAstV-1 | MG932591 | Russia | Europe | 2017 |
| HAstV-1 | MH446377 | Russia | Europe | 2018 |
| HAstV-1 | MH446378 | Russia | Europe | 2018 |
| HAstV-1 | MH446380 | Russia | Europe | 2018 |
| HAstV-1 | MH446382 | Russia | Europe | 2018 |
| HAstV-1 | AY093652 | South Africa | Africa | 1998 |
| HAstV-1 | AY093654 | South Africa | Africa | 1999 |
| HAstV-1 | AY093655 | South Africa | Africa | 1999 |
| HAstV-1 | AY094080 | South Africa | Africa | 1999 |
| HAstV-1 | AY094082 | South Africa | Africa | 1999 |
| HAstV-1 | AY094086 | South Africa | Africa | 1999 |
| HAstV-1 | AY094088 | South Africa | Africa | 1999 |
| HAstV-1 | AY094081 | South Africa | Africa | 2000 |
| HAstV-1 | KP636520 | Tunisia | Africa | 2011 |
| HAstV-1 | KP636521 | Tunisia | Africa | 2011 |
| HAstV-1 | KP636522 | Tunisia | Africa | 2011 |
| HAstV-1 | KP636523 | Tunisia | Africa | 2011 |
| HAstV-1 | KP636524 | Tunisia | Africa | 2011 |
| HAstV-1 | KP636525 | Tunisia | Africa | 2011 |
| HAstV-1 | AB000291 | UK | Europe | 1990 |
| HAstV-1 | AB000289 | UK | Europe | 1993 |
| HAstV-1 | L23513 | UK | Europe | 1993 |
| HAstV-1 | Z25771 | UK | Europe | 1993 |
| HAstV-1 | AB000283 | UK | Europe | 1997 |
| HAstV-1 | EF519313 | UK | Europe | 2006 |
| HAstV-1 | KP064286 | Uruguay | South America | 2011 |
| HAstV-1 | KP064288 | Uruguay | South America | 2012 |
| HAstV-1 | KP064289 | Uruguay | South America | 2012 |
| HAstV-1 | KP064290 | Uruguay | South America | 2012 |
| HAstV-1 | KP064291 | Uruguay | South America | 2012 |
| HAstV-1 | KP064292 | Uruguay | South America | 2012 |
| HAstV-1 | KP064293 | Uruguay | South America | 2012 |
| HAstV-1 | KP064294 | Uruguay | South America | 2012 |
| HAstV-1 | KP064295 | Uruguay | South America | 2013 |
| HAstV-1 | KP064297 | Uruguay | South America | 2013 |
| HAstV-1 | AY304451 | USA | North America | 1998 |
| HAstV-1 | AY304455 | USA | North America | 1998 |
| HAstV-1 | AY304456 | USA | North America | 1998 |
| HAstV-1 | AY304457 | USA | North America | 1998 |
| HAstV-1 | AY304458 | USA | North America | 1998 |
| HAstV-1 | AY304452 | USA | North America | 1999 |
| HAstV-1 | AY304453 | USA | North America | 1999 |
| HAstV-1 | AY304454 | USA | North America | 1999 |
| HAstV-1 | AY304459 | USA | North America | 1999 |
| HAstV-1 | AY304460 | USA | North America | 1999 |
| HAstV-1 | GU248213 | Venezuela | South America | 2003 |
| HAstV-1 | GU248214 | Venezuela | South America | 2003 |
| HAstV-2 | KM269060 | Brazil | South America | 2005 |
| HAstV-2 | KM269061 | Brazil | South America | 2006 |
| HAstV-2 | KM269062 | Brazil | South America | 2007 |
| HAstV-2 | KP862744 | Korea | Asia | 2014 |
| HAstV-2 | MG557598 | Mexico | North America | 2013 |
| HAstV-2 | MG557599 | Mexico | North America | 2013 |
| HAstV-2 | MG557600 | Mexico | North America | 2013 |
| HAstV-2 | KF039910 | Russia | Europe | 2005 |
| HAstV-2 | KF039911 | Russia | Europe | 2006 |
| HAstV-2 | MG924483 | Russia | Europe | 2008 |
| HAstV-2 | KC285152 | Russia | Europe | 2011 |
| HAstV-2 | KC285160 | Russia | Europe | 2011 |
| HAstV-2 | KP208781 | Russia | Europe | 2013 |
| HAstV-2 | KT749761 | Russia | Europe | 2014 |
| HAstV-2 | KT749762 | Russia | Europe | 2015 |
| HAstV-2 | KP064287 | Uruguay | South America | 2011 |
| HAstV-3 | AY324864 | Argentina | South America | 1995.7-1998.10 |
| HAstV-3 | AY324865 | Argentina | South America | 1995.7-1998.10 |
| HAstV-3 | KC137247 | Australia | Oceania | 2009 |
| HAstV-3 | KF420153 | Bangladesh | Asia | 2012 |
| HAstV-3 | DQ917402 | Brazil | South America | 1991 |
| HAstV-3 | DQ917403 | Brazil | South America | 1991 |
| HAstV-3 | DQ917400 | Brazil | South America | 1992 |
| HAstV-3 | DQ917401 | Brazil | South America | 1992 |
| HAstV-3 | DQ071649 | Brazil | South America | 1997 |
| HAstV-3 | KM269066 | Brazil | South America | 2007 |
| HAstV-3 | KM269063 | Brazil | South America | 2008 |
| HAstV-3 | KM269064 | Brazil | South America | 2008 |
| HAstV-3 | KM269065 | Brazil | South America | 2009 |
| HAstV-3 | KP208157 | Brazil | South America | 2010 |
| HAstV-3 | KP162255 | Bulgaria | Europe | 2009 |
| HAstV-3 | GU384243 | China | Asia | 2005 |
| HAstV-3 | GU384249 | China | Asia | 2006 |
| HAstV-3 | GQ169036 | China | Asia | 2007 |
| HAstV-3 | DQ630763 | China | Asia | 2007 |
| HAstV-3 | AF211958 | Colombia | South America | 1997.06-1999.05 |
| HAstV-3 | HQ674643 | Egypt | Africa | 2007 |
| HAstV-3 | AY007585 | Germany | Europe | 1998 |
| HAstV-3 | AY007586 | Germany | Europe | 1998 |
| HAstV-3 | AY007587 | Germany | Europe | 1999 |
| HAstV-3 | AY007588 | Germany | Europe | 1999 |
| HAstV-3 | AF395735 | Hungary | Europe | 1998 |
| HAstV-3 | AJ620760 | Hungary | Europe | 2002 |
| HAstV-3 | AB548404 | India | Asia | 2009 |
| HAstV-3 | AB551378 | India | Asia | 2009 |
| HAstV-3 | MK296753 | Ireland | Europe | 2018 |
| HAstV-3 | HM212542 | Japan | Asia | 2009 |
| HAstV-3 | KF453630 | Pakistan | Asia | 2008 |
| HAstV-3 | GU223905 | Russia | Europe | 2003 |
| HAstV-3 | JF491366 | Russia | Europe | 2004 |
| HAstV-3 | JF491430 | Russia | Europe | 2004 |
| HAstV-3 | JF491380 | Russia | Europe | 2005 |
| HAstV-3 | GU732187 | Russia | Europe | 2008 |
| HAstV-3 | JF929194 | Russia | Europe | 2009 |
| HAstV-3 | MH332779 | Russia | Europe | 2010 |
| HAstV-3 | KC285117 | Russia | Europe | 2010 |
| HAstV-3 | KC285119 | Russia | Europe | 2010 |
| HAstV-3 | MH332782 | Russia | Europe | 2011 |
| HAstV-3 | MH332784 | Russia | Europe | 2011 |
| HAstV-3 | MH332785 | Russia | Europe | 2011 |
| HAstV-3 | AY093650 | South Africa | Africa | 1998 |
| HAstV-3 | AY094087 | South Africa | Africa | 1999 |
| HAstV-3 | AY094090 | South Africa | Africa | 1999 |
| HAstV-3 | JQ045382 | Tunisia | Africa | 2010 |
| HAstV-3 | JQ045383 | Tunisia | Africa | 2010 |
| HAstV-3 | AY304465 | USA | North America | 1998 |
| HAstV-3 | AY304464 | USA | North America | 1999 |
| HAstV-3 | GU248215 | Venezuela | South America | 2003 |
| HAstV-3 | MG571777 | Venezuela | South America | 2015 |
| HAstV-4 | AY324854 | Argentina | South America | 1995.7-1998.10 |
| HAstV-4 | AY324855 | Argentina | South America | 1995.7-1998.10 |
| HAstV-4 | AY324856 | Argentina | South America | 1995.7-1998.10 |
| HAstV-4 | KC137248 | Australia | Oceania | 2010 |
| HAstV-4 | KC137249 | Australia | Oceania | 2010 |
| HAstV-4 | U49218 | Australia | Oceania | 1995 |
| HAstV-4 | U49219 | Australia | Oceania | 1995 |
| HAstV-4 | AF175259 | Australia | Oceania | 1998 |
| HAstV-4 | DQ917404 | Brazil | South America | 1990 |
| HAstV-4 | DQ917405 | Brazil | South America | 1990 |
| HAstV-4 | DQ917406 | Brazil | South America | 1990 |
| HAstV-4 | DQ917407 | Brazil | South America | 1990 |
| HAstV-4 | DQ070852 | Brazil | South America | 1995 |
| HAstV-4 | DQ071655 | Brazil | South America | 1999 |
| HAstV-4 | AY846635 | Brazil | South America | 1999 |
| HAstV-4 | DQ381505 | Brazil | South America | 2004 |
| HAstV-4 | KM269067 | Brazil | South America | 2006 |
| HAstV-4 | KM459014 | Brazil | South America | 2010 |
| HAstV-4 | GU384258 | China | Asia | 2005 |
| HAstV-4 | DQ344027 | China | Asia | 2005 |
| HAstV-4 | GQ405857 | China | Asia | 2007 |
| HAstV-4 | KY828138 | China | Asia | 2015 |
| HAstV-4 | KY828137 | China | Asia | 2016 |
| HAstV-4 | KY828139 | China | Asia | 2016 |
| HAstV-4 | AF211957 | Colombia | South America | 1997.06-1999.02 |
| HAstV-4 | HQ674632 | Egypt | Africa | 2007 |
| HAstV-4 | HQ674633 | Egypt | Africa | 2007 |
| HAstV-4 | AY007589 | Germany | Europe | 1998 |
| HAstV-4 | AY007590 | Germany | Europe | 1999 |
| HAstV-4 | AY007591 | Germany | Europe | 1999 |
| HAstV-4 | AY720891 | Germany | Europe | 2004 |
| HAstV-4 | AF395736 | Hungary | Europe | 1995.3-1999.2 |
| HAstV-4 | AJ620753 | Hungary | Europe | 2002 |
| HAstV-4 | AJ620754 | Hungary | Europe | 2002 |
| HAstV-4 | AJ620758 | Hungary | Europe | 2002 |
| HAstV-4 | GU216586 | Italy | Europe | 2002 |
| HAstV-4 | GU216587 | Italy | Europe | 2002 |
| HAstV-4 | GU216588 | Italy | Europe | 2002 |
| HAstV-4 | JQ434395 | Italy | Europe | 2008 |
| HAstV-4 | JQ434396 | Italy | Europe | 2008 |
| HAstV-4 | AB025812 | Japan | Asia | 1987 |
| HAstV-4 | AB025802 | Japan | Asia | 1988 |
| HAstV-4 | AB025803 | Japan | Asia | 1988 |
| HAstV-4 | AB025811 | Japan | Asia | 1990 |
| HAstV-4 | AB025801 | Japan | Asia | 1991 |
| HAstV-4 | AB025810 | Japan | Asia | 1992 |
| HAstV-4 | AB025804 | Japan | Asia | 1993 |
| HAstV-4 | AB025805 | Japan | Asia | 1993 |
| HAstV-4 | AB025806 | Japan | Asia | 1993 |
| HAstV-4 | AB025807 | Japan | Asia | 1993 |
| HAstV-4 | AB025808 | Japan | Asia | 1993 |
| HAstV-4 | AB025809 | Japan | Asia | 1993 |
| HAstV-4 | KF039912 | Russia | Europe | 2005 |
| HAstV-4 | JF491382 | Russia | Europe | 2005 |
| HAstV-4 | GU212646 | Russia | Europe | 2006 |
| HAstV-4 | GU212647 | Russia | Europe | 2006 |
| HAstV-4 | JF929195 | Russia | Europe | 2009 |
| HAstV-4 | MH332778 | Russia | Europe | 2010 |
| HAstV-4 | KF039913 | Russia | Europe | 2010 |
| HAstV-4 | KC285110 | Russia | Europe | 2010 |
| HAstV-4 | KC285113 | Russia | Europe | 2010 |
| HAstV-4 | JF491420 | Russia | Europe | 2010 |
| HAstV-4 | MG932573 | Russia | Europe | 2012 |
| HAstV-4 | MG932576 | Russia | Europe | 2012 |
| HAstV-4 | MG932579 | Russia | Europe | 2016 |
| HAstV-4 | KY294663 | Russia | Europe | 2016 |
| HAstV-4 | KY294665 | Russia | Europe | 2016 |
| HAstV-4 | KY294666 | Russia | Europe | 2016 |
| HAstV-4 | KY294667 | Russia | Europe | 2016 |
| HAstV-4 | KY294668 | Russia | Europe | 2016 |
| HAstV-4 | KY294669 | Russia | Europe | 2016 |
| HAstV-4 | KY294670 | Russia | Europe | 2016 |
| HAstV-4 | MG932580 | Russia | Europe | 2017 |
| HAstV-4 | MG932581 | Russia | Europe | 2017 |
| HAstV-4 | MG932584 | Russia | Europe | 2017 |
| HAstV-4 | MG932586 | Russia | Europe | 2017 |
| HAstV-4 | MG932587 | Russia | Europe | 2017 |
| HAstV-4 | AY094092 | South Africa | Africa | 1999 |
| HAstV-4 | AB496913 | Spain | Europe | 2009 |
| HAstV-4 | Z33883 | UK | Europe | 1971 |
| HAstV-4 | AY304466 | USA | North America | 1998 |
| HAstV-5 | AY324866 | Argentina | South America | 1995.7-1998.10 |
| HAstV-5 | AY324867 | Argentina | South America | 1995.7-1998.10 |
| HAstV-5 | AY324868 | Argentina | South America | 1995.7-1998.10 |
| HAstV-5 | U49220 | Australia | Oceania | 1995 |
| HAstV-5 | KF420150 | Bangladesh | Asia | 2012 |
| HAstV-5 | KF420151 | Bangladesh | Asia | 2012 |
| HAstV-5 | KF420152 | Bangladesh | Asia | 2012 |
| HAstV-5 | DQ028633 | Brazil | South America | 1994 |
| HAstV-5 | DQ071650 | Brazil | South America | 1998 |
| HAstV-5 | AB037273 | China | Asia | 1996 |
| HAstV-5 | AB037274 | China | Asia | 1996 |
| HAstV-5 | JF343978 | China | Asia | 2009 |
| HAstV-5 | JQ403108 | China | Asia | 2010 |
| HAstV-5 | MF684776 | China | Asia | 2013 |
| HAstV-5 | KJ935465 | China | Asia | 2013 |
| HAstV-5 | KJ935466 | China | Asia | 2013 |
| HAstV-5 | KJ935467 | China | Asia | 2013 |
| HAstV-5 | KJ935468 | China | Asia | 2013 |
| HAstV-5 | KY311992 | China | Asia | 2015 |
| HAstV-5 | KC223582 | France | Europe | 2012 |
| HAstV-5 | EU327560 | Ghana | Africa | 2006 |
| HAstV-5 | AF395737 | Hungary | Europe | 1995.03-1999.02 |
| HAstV-5 | KF157967 | Hungary | Europe | 2012 |
| HAstV-5 | JQ434397 | Italy | Europe | 2008 |
| HAstV-5 | AB769838 | Japan | Asia | 1976 |
| HAstV-5 | KY905157 | Korea | Asia | 2014 |
| HAstV-5 | KY200611 | Morocco | Africa | 2012 |
| HAstV-5 | JF929196 | Russia | Europe | 2010 |
| HAstV-5 | JF929197 | Russia | Europe | 2010 |
| HAstV-5 | JF929198 | Russia | Europe | 2010 |
| HAstV-5 | JF929199 | Russia | Europe | 2010 |
| HAstV-5 | MH332787 | Russia | Europe | 2011 |
| HAstV-5 | JN203052 | Russia | Europe | 2011 |
| HAstV-5 | MG932569 | Russia | Europe | 2012 |
| HAstV-5 | MG932570 | Russia | Europe | 2012 |
| HAstV-5 | MG932571 | Russia | Europe | 2012 |
| HAstV-5 | MG932572 | Russia | Europe | 2012 |
| HAstV-5 | MG932574 | Russia | Europe | 2012 |
| HAstV-5 | MG932575 | Russia | Europe | 2012 |
| HAstV-5 | KT749763 | Russia | Europe | 2012 |
| HAstV-5 | KT749765 | Russia | Europe | 2012 |
| HAstV-5 | KM923915 | Russia | Europe | 2012 |
| HAstV-5 | KF039918 | Russia | Europe | 2012 |
| HAstV-5 | KF039920 | Russia | Europe | 2012 |
| HAstV-5 | KC285177 | Russia | Europe | 2012 |
| HAstV-5 | KC285178 | Russia | Europe | 2012 |
| HAstV-5 | KC285187 | Russia | Europe | 2012 |
| HAstV-5 | KT749764 | Russia | Europe | 2013 |
| HAstV-5 | KT749766 | Russia | Europe | 2013 |
| HAstV-5 | AY093651 | South Africa | Africa | 1998 |
| HAstV-5 | AY094089 | South Africa | Africa | 1999 |
| HAstV-5 | KP064296 | Uruguay | South America | 2013 |
| HAstV-5 | U15136 | USA | North America | 1994 |
| HAstV-5 | AY304467 | USA | North America | 1999 |
| HAstV-6 | KF420154 | Bangladesh | Asia | 2011 |
| HAstV-6 | DQ917382 | Brazil | South America | 1991 |
| HAstV-6 | KM269069 | Brazil | South America | 2006 |
| HAstV-6 | KM269068 | Brazil | South America | 2010 |
| HAstV-6 | GQ495608 | China | Asia | 2007 |
| HAstV-6 | FJ755390 | China | Asia | 2007 |
| HAstV-6 | EU327559 | Ghana | Africa | 2006 |
| HAstV-6 | HM237363 | Japan | Asia | 2010 |
| HAstV-6 | MG557601 | Mexico | North America | 2013 |
| HAstV-6 | GQ901902 | Russia | Europe | 2009 |
| HAstV-6 | AY093653 | South Africa | Africa | 1998 |
| HAstV-6 | Z46658 | UK | Europe | 1989 |
| HAstV-7 | AB00300 | Japan | Asia | 1997 |
| HAstV-7 | Y08632 | Norway | Europe | 1996 |
| HAstV-7 | AY094091 | South Africa | Africa | 1999 |
| HAstV-7 | AF248738 | UK | Europe | 1991 |
| HAstV-7 | AY304468 | USA | North America | 1999 |
| HAstV-8 | AF175261 | Australia | Oceania | 1997 |
| HAstV-8 | DQ990460 | Brazil | South America | 1993 |
| HAstV-8 | DQ381506 | Brazil | South America | 2004 |
| HAstV-8 | DQ381507 | Brazil | South America | 2004 |
| HAstV-8 | DQ381508 | Brazil | South America | 2004 |
| HAstV-8 | KM269070 | Brazil | South America | 2006 |
| HAstV-8 | KJ935469 | China | Asia | 2013 |
| HAstV-8 | HQ674644 | Egypt | Africa | 2007 |
| HAstV-8 | EU327562 | Ghana | Africa | 2006 |
| HAstV-8 | AF395738 | Hungary | Europe | 1995.03-1999.02 |
| HAstV-8 | AJ620757 | Hungary | Europe | 2002 |
| HAstV-8 | MF818323 | India | Asia | 2010 |
| HAstV-8 | MF818324 | India | Asia | 2014 |
| HAstV-8 | MF818325 | India | Asia | 2014 |
| HAstV-8 | KY815038 | India | Asia | 2015 |
| HAstV-8 | MF818326 | India | Asia | 2015 |
| HAstV-8 | AB693980 | Japan | Asia | 2008 |
| HAstV-8 | AB476427 | Japan | Asia | 2008 |
| HAstV-8 | MK472055 | Korea | Asia | 2014 |
| HAstV-8 | MK472056 | Korea | Asia | 2014 |
| HAstV-8 | KP862744 | Korea | Asia | 2014 |
| HAstV-8 | AF260508 | Mexico | North America | 2000 |
| HAstV-8 | KP015059 | Netherlands | Europe | 2012 |
| HAstV-8 | KP015060 | Netherlands | Europe | 2012 |
| HAstV-8 | KP015061 | Netherlands | Europe | 2012 |
| HAstV-8 | KP015062 | Netherlands | Europe | 2012 |
| HAstV-8 | KP015063 | Netherlands | Europe | 2012 |
| HAstV-8 | KP015064 | Netherlands | Europe | 2012 |
| HAstV-8 | KP015065 | Netherlands | Europe | 2012 |
| HAstV-8 | KP015066 | Netherlands | Europe | 2012 |
| HAstV-8 | KP015067 | Netherlands | Europe | 2012 |
| HAstV-8 | KM923916 | Russia | Europe | 2012 |
| HAstV-8 | KY294664 | Russia | Europe | 2016 |
| HAstV-8 | AY093649 | South Africa | Africa | 1998 |
| HAstV-8 | AY094083 | South Africa | Africa | 2000 |
| HAstV-8 | Z66541 | UK | Europe | 1995 |
| HAstV-8 | AY304469 | USA | North America | 1998 |
| HAstV-8 | AY304470 | USA | North America | 1999 |
