# Supplementary material for: Molecular Evolution of Classic Human Astrovirus, as Revealed by the Analysis of the Capsid Protein Gene
Source: Viruses. 2019 Aug 1;11(8):707. doi: 10.3390/v11080707 (PMC6722597; doi:10.3390/v11080707)
Supplement: Supplementary file 1 [file viruses-11-00707-s001.zip › Table S2.docx]

**Table S2 Complete sequences of classic HAstV ORF2 gene used in the study**

| KC342249/HAstV-1/km1/CHN/2012 |
| --- |
| KF211475/HAstV-1/JZ/CHN/2010 |
| KY744141/HAstV-1/PA387/ITA/2011 |
| KY744140/HAstV-1/PA148/ITA/2008 |
| KY744139/HAstV-1/PA70R/ITA/2004 |
| KY744138/HAstV-1/PA364/ITA/1999 |
| KY744137/HAstV-1/PA762/ITA/2000 |
| HQ398856/HAstV-1/Nyergesujfalu/HUN4520/HUN/2010 |
| KP942593/HAstV-1/KMSP_12/CHN/2013 |
| KP942592/HAstV-1/KMSP_11/CHN/2013 |
| KP942591/HAstV-1/KMSP_10/CHN/2013 |
| KP942590/HAstV-1/KMSP_09/CHN/2013 |
| KP942589/HAstV-1/KMSP_08/CHN/2014 |
| KP942588/HAstV-1/KMSP_06/CHN/2014 |
| KP942587/HAstV-1/KMSP_05/CHN/2014 |
| KP942586/HAstV-1/KMSP_04/CHN/2014 |
| KP942585/HAstV-1/KMSP_03/CHN/2014 |
| KP942584/HAstV-1/KMSP_02/CHN/2014 |
| KP942583/HAstV-1/KMSP_01/CHN/2014 |
| KP942582/HAstV-1/KMNP_00/2014 |
| JX087965/HAstV-1/PA124/ITA/2005 |
| JN887820/HAstV-1/lhar/KR/2011 |
| FJ792842/HAstV-1/Shanghai/CHN/2008 |
| GQ405856/HAstV-1/Shenyang/CHN/2007 |
| GQ405855/HAstV-1/Dalian/CHN/2007 |
| AB009985/HAstV-1/J1050/JPN/1993 |
| AB009984/HAstV-1/J153/JPN/1992 |
| AB000287/HAstV-1/JAPAS_115/JPN/1992 |
| AB000286/HAstV-1/816/JPN/1993 |
| AB000285/HAstV-1/P437/PAK/1991 |
| AB000284/HAstV-1/JAPAS_1526/JPN/1992 |
| AB000283/HAstV-1/S1/UK/1997 |
| AY720892/HAstV-1/Dresden/GER/2004 |
| L23513/HAstV-1/Oxford/UK/1993 |
| Z25771/HAstV-1/A2-88Newcastle/UK/1993 |
| FJ755405/HAstV-1/Beijing/293/CHN/2007 |
| FJ755404/HAstV-1/Beijing/291/CHN/2007 |
| FJ755403/HAstV-1/Beijing/176/CHN/2006 |
| FJ755402/HAstV-1/Beijing/128/CHN/2005 |
| KY271945/HAstV-1/TN/2015-OB2038A/USA/2015 |
| EF138826/HAstV-1/3085/USA/1999 |
| EF138825/HAstV-1/2987/USA/1999 |
| EF138824/HAstV-1/2882/USA/1999 |
| EF138823/HAstV-1/1638/USA/1999 |
| FJ375759/HAstV-1/SH1/CHN/2008 |
| JF327666/HAstV-1/Pune/063681/IND/2006 |
| JX087964/HAstV-2/PR5142/ITA/2009 |
| JX087963/HAstV-2/PA65R/ITA/2002 |
| L06802/HAstV-2/Oxford/UK/1993 |
| EF138827/HAstV-2/1299/USA/1999 |
| L13745/HAstV-2/Oxford/1993 |
| AB000290/HAstV-2/NORAS_1128/NOR/1993 |
| MG571777/HAstV-3/V1A/VEN/2015 |
| JF491430/HAstV-3/Rus-Nsc04-H355/RUS/2004 |
| GU732187/HAstV-3/Rus-Nsc08-3364/2008 |
| GU223905/HAstV-3/Rus-Nsc03-H191/RUS/2003 |
| DQ630763/HAstV-3/WH1859/CHN/2004 |
| AF117209/HAstV-3/H3/USA/1998 |
| EF138829/HAstV-3/3207/USA/1999 |
| EF138828/HAstV-3/754/USA/1999 |
| KY271946/HAstV-3/TN/2015-OB2038B/USA/2015 |
| AF141381/HAstV-3/Berlin/GER/1999 |
| KF668570/HAstV-3/PR1365/ITA/2012 |
| AB000295/HAstV-3/O-35/JPN/1993 |
| AB000294/HAstV-3/O-28/JPN/1993 |
| AB000293/HAstV-3/O-14/JPN/1993 |
| AB000292/HAstV-3/O-13/JPN/1993 |
| AB000291/HAstV-3/S3/UK/1990 |
| KC915035/HAstV-4/PA73/ITA/2003 |
| KC915034/HAstV-4/BA393/08-65_/ITA/2008 |
| AY720891/HAstV-4/Dresden/GER/2004 |
| DQ344027/HAstV-4/Guangzhou/2004 |
| DQ070852/HAstV-4/Goiania/GO/12/BRA/1995 |
| GQ405857/HAstV-4/Panjin/CHN/2007 |
| AB496913/HAstV-4/ESP/2009 |
| AB025812/HAstV-4/O-33/87-25/Ehime1987/JPN/1987 |
| AB025811/HAstV-4/O-29/90-276/Ehime1990/JPN/1990 |
| AB025810/HAstV-4/O-27/92-676/Ehime1992/JPN/1992 |
| AB025809/HAstV-4/O-25/93-197/Ehime1993/JPN/1993 |
| AB025808/HAstV-4/O-24/93-439/Ehime1993/JPN/1993 |
| AB025807/HAstV-4/O-23/93-482/Ehime1993/JPN/1993 |
| AB025806/HAstV-4/O-22/93-525/Ehime1993/JPN/1993 |
| AB025805/HAstV-4/O-21/93-583/Ehime1993/JPN/1993 |
| AB025804/HAstV-4/O-20/93-601/Ehime1993/JPN/1993 |
| AB025803/HAstV-4/O-16/88-561/Ehime1988/JPN/1988 |
| AB025802/HAstV-4/O-15/88-532/Ehime1988/JPN/1988 |
| AB025801/HAstV-4/O-03/91-283/Ehime1991/JPN/1991 |
| Z33883/HAstV-4/Oxford-H4/UK/1971 |
| JQ403108/HAstV-5/DL030/CHN/2010 |
| MF684776/HAstV-5/Fuzhou/85/CHN/2013 |
| DQ028633/HAstV-5/Goiania/GO/12/BRA/1994 |
| KF157967/HAstV-5/Budapest/HUN5186/HUN/2012 |
| AB037274/HAstV-5/CHN198/1996 |
| AB037273/HAstV-5/CHN146/CHN/1996 |
| U15136/HAstV-5/Oxford-5/USA/1994 |
| MH933759/HAstV-5/CMRHP43/CMR/2014 |
| MH933758/HAstV-5/CMRHP34/CMR/2014 |
| AB000298/HAstV-5/O-1/JPN/1993 |
| AB013618/HAstV-6/Osaka-91/JPN/1991 |
| AB031030/HAstV-6/katano-24/JPN/1999 |
| AB031031/HAstV-6/katano-23-6/JPN/1999 |
| GQ901902/HAstV-6/Rus-Nsc09-B4/RUS/2009 |
| HM237363/HAstV-6/katano/JPN/2010 |
| GQ495608/HAstV-6/192-BJ07/CHN/2007 |
| Z46658/HAstV-6/Oxford-6/UK/1989 |
| AF248738/HAstV-7/Oxford-7/UK/1991 |
| Y08632/HAstV-7/NOR/1996 |
| AB000300/HAstV-7/S7/JPN/1997 |
| AF260508/HAstV-8/Yuc-8/MEX/2000 |
| Z66541/HAstV-8/H8/UK/1995 |
| EF138831/HAstV-8/3383/USA/1999 |
| EF138830/HAstV-8/2701/USA/1999 |
| AB000301/HAstV-8/SX_PAKAS_706/PAK/1993 |
| MH933757/HAstV-8/CMRHP35D/CMR/2014 |
| MH933753/HAstV-8/CMRHP3/CMR/2014 |
| MH933752/HAstV-8/CMRHP2/CMR/2014 |
